# Characterization of Novel Sorghum *brown midrib* Mutants from an EMS-Mutagenized Population

**DOI:** 10.1534/g3.114.014001

**Published:** 2014-09-02

**Authors:** Scott E. Sattler, Ana Saballos, Zhanguo Xin, Deanna L. Funnell-Harris, Wilfred Vermerris, Jeffrey F. Pedersen

**Affiliations:** * Grain, Forage and Bioenergy Research Unit, USDA-ARS, Lincoln, Nebraska 68583; † Department of Agronomy and Horticulture, University of Nebraska–Lincoln, Lincoln, Nebraska 68583; ‡ Agronomy Department and Genetics Institute, University of Florida, Gainesville, Florida 32610; § Plant Stress and Germplasm Development Unit, USDA-ARS, Lubbock, Texas 79415; ** Department of Plant Pathology, University of Nebraska–Lincoln, Lincoln, Nebraska 68583; †† Department of Microbiology and Cell Science and Genetics Institute, University of Florida, Gainesville, Florida 32610

**Keywords:** acid detergent lignin (ADL), ethyl methanesulfonate (EMS), bioenergy, forage composition, allelism

## Abstract

Reducing lignin concentration in lignocellulosic biomass can increase forage digestibility for ruminant livestock and saccharification yields of biomass for bioenergy. In sorghum (*Sorghum bicolor* (L.) Moench) and several other C4 grasses, *brown midrib* (*bmr*) mutants have been shown to reduce lignin concentration. Putative *bmr* mutants isolated from an EMS-mutagenized population were characterized and classified based on their leaf midrib phenotype and allelism tests with the previously described sorghum *bmr* mutants *bmr2*, *bmr6*, and *bmr12*. These tests resulted in the identification of additional alleles of *bmr2*, *bmr6*, and *bmr12*, and, in addition, six *bmr* mutants were identified that were not allelic to these previously described loci. Further allelism testing among these six *bmr* mutants showed that they represented four novel *bmr* loci. Based on this study, the number of *bmr* loci uncovered in sorghum has doubled. The impact of these lines on agronomic traits and lignocellulosic composition was assessed in a 2-yr field study. Overall, most of the identified *bmr* lines showed reduced lignin concentration of their biomass relative to wild-type (WT). Effects of the six new *bmr* mutants on enzymatic saccharification of lignocellulosic materials were determined, but the amount of glucose released from the stover was similar to WT in all cases. Like *bmr2*, *bmr6*, and *bmr12*, these mutants may affect monolignol biosynthesis and may be useful for bioenergy and forage improvement when stacked together or in combination with the three previously described *bmr* alleles.

Plant cell walls are vast reserves of photosynthetically fixed carbon. Global research efforts are focused on utilizing plant cell walls as renewable resources for the production of energy, chemical precursors, and fuels; however, the traditional uses of plant cell walls as fodder and fiber also remain important. Plant cell walls predominantly consist of the polysaccharides cellulose and hemicellulose and the phenolic polymer lignin. Lignin accounts for approximately 30% of organic carbon in the biosphere (Boerjan *et al.* 2003), and its environmental abundance is exceeded only by cellulose (Jung and Ni 1998). The lignin polymer cross-links cell wall polysaccharides, thereby stiffening and reinforcing the secondary cell wall structure (Boerjan *et al.* 2003). The resulting matrix is recalcitrant to both chemical degradation and biological digestion, which impairs hydrolysis of the polysaccharides into their monomeric sugars in ruminant livestock or cellulosic bioenergy systems. Hence, reducing lignin has become an important target for both bioenergy feedstock improvement (Chen and Dixon 2007; Dien *et al.* 2009; Vermerris *et al.* 2007) and improving fodder digestibility (Barrière *et al.* 2003; Guo *et al.* 2001; Jung and Allen 1995; Vogel and Jung 2001). Although lignin makes liberating sugars from cell walls more difficult, lignin serves critical functions for vascular plants. Lignin is required for vascular elements to transport water under negative pressure, a critical adaptive feature that allowed vascular plants to colonize land during their evolution (Boyce *et al.* 2004; Sperry 2003). The collapse of vascular elements has been observed in mutants extremely impaired in lignin synthesis (Jones *et al.* 2001; Piquemal *et al.* 1998; Ruel *et al.* 2009). Hence, there is a limit to the extent lignin content may be manipulated without significantly impacting plant fitness.

The subunits of lignin are synthesized from the amino acid phenylalanine, and aromatic amino acid synthesis and phenylpropanoid metabolism play central roles in vascular plants. Approximately 30% of the carbon flux passes through phenylalanine in plants (Tohge *et al.* 2013), which illustrates the importance of this pathway. In angiosperms, there are three main subunits of lignin [*p*-hydroxyphenol (H), guaiacyl (G), syringyl (S)] that are polymerized within the cell wall from their respective monolignols (*p*-coumaryl, coniferyl, sinapyl alcohols) via oxidative coupling (Boerjan *et al.* 2003). A consensus model of the monolignol biosynthetic pathway (Vanholme *et al.* 2013) has been developed based on the identification and characterization of monolignol biosynthetic enzymes from a range of plant species and the amino acid sequence conservation of these enzymes across plant genomes (Figure 1).

**Figure 1:**
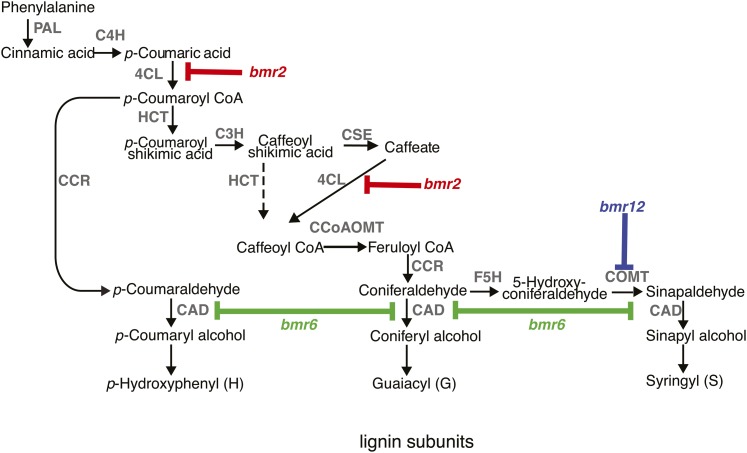
The monolignol biosynthetic pathway in sorghum based on consensus models from dicot and monocot plants (Vanholme *et al.* 2013). The enzymatic steps (gray) are as follows: phenylalanine ammonia lyase (PAL); cinnamate 4-hydroxylase (C4H); 4-coumarate-CoA ligase (4CL); hydroxycinnamoyl CoA:shikimate transferase (HCT); *p*-coumarate 3-hydroxylase (C3H); caffeoyl shikimate esterase (CSE); caffeoyl CoA *O*-methyltransferase (CCoAOMT); cinnamyl CoA reductase (CRR); ferulate 5-hydroxylase (F5H); caffeic acid *O*-methyltransferase (COMT); and cinnamyl alcohol dehydrogenase (CAD). The *bmr2*, *bmr6*, and *bmr12* mutants are impaired in 4CL, COMT, and CAD enzymatic activities, respectively.

The *brown midrib* phenotype has been useful for identifying mutants impaired in lignin synthesis in C4 grasses (Kuc and Nelson 1964; Gee *et al.* 1968; Porter *et al.* 1978; Cherney *et al.* 1988), because the tan to reddish brown leaf midribs visibly contrast to the white or green midribs observed in wild-type (WT) plants. In maize (*Zea mays*), spontaneous *brown midrib* (*bm*) mutants were first identified in the 1920s (Jorgenson 1931). To date, there are six known *brown midrib* (*bm1-6*) loci that have been identified in maize (Ali *et al.* 2010). In sorghum (*Sorghum bicolor*), a series of *brown midrib* (*bmr*) mutants was isolated from diethyl sulfate (DES) mutagenized populations at Purdue University in the 1970s (Porter *et al.* 1978; Bittinger *et al.* 1981). Allelism tests from this series identified four sorghum *bmr* loci, *bmr2*, *bmr6*, *bmr12*, and *bmr19* (Saballos *et al.* 2008). The *bmr19* mutant is not publicly available (effectively reducing the available sorghum *brown midrib* mutants to a set of three independent loci: *bmr2*, *bmr6*, and *bmr12*). However, *bmr19* appears to be of limited value for forage and bioenergy applications, because it did not significantly reduce lignin concentration and did not markedly alter lignin subunit composition (Saballos *et al.* 2008). *Brown midrib* mutants have also been isolated in pearl millet (*Pennisetum glaucum*) (Cherney *et al.* 1988; Degenhart *et al.* 1995; Gupta 1995).

The *brown midrib* mutants have been used to identify and characterize the genes that encode the major enzymes for specific steps of monolignol biosynthesis for C4 grasses (Figure 1). The maize *Bm3* and sorghum *Bmr12* genes both encode orthologous caffeic acid *O*-methyltransferase (COMT), which catalyzes the penultimate step in monolignol biosynthesis (Vignols *et al.* 1995; Bout and Vermerris 2003). The maize *Bm1* and the sorghum *Bmr*6 genes both encode orthologous cinnamyl alcohol dehydrogenase (CAD) (Saballos *et al.* 2009; Sattler *et al.* 2009; Chen *et al.* 2012), which catalyzes the last step in monolignol biosynthesis. *Bmr2* was shown to encode a 4-coumarate coenzyme A ligase (4CL), which catalyzes an early step in monolignol biosynthesis (Saballos *et al.* 2012). Recently, the maize *Bm2* gene was cloned and shown to encode a methylenetetrahydrofolate reductase (Tang *et al.* 2014). This enzyme catalyzes the rate-limiting step in one carbon (folate) metabolism and for the synthesis of the methyl donor *S*-adenosyl-L-methionine (SAM) (Roje *et al.* 1999, 2002). SAM is a cofactor for two methylation reactions in monolignol biosynthesis catalyzed by the caffeoyl-CoA *O*-methyltransferase (CCoAOMT) and the caffeic acid *O*-methyltransferase (COMT), respectively (Figure 1). The maize *Bm4*, *Bm5*, and *Bm6* and sorghum *Bmr19* genes have not yet been cloned and their functions remain to be fully elucidated.

Based on examination of lignin biosynthesis pathways, there is clear potential for major perturbations of monolignol synthesis at key enzymatic steps, any of which could result in reduced lignin concentration and/or altered lignin composition associated with the *brown midrib* phenotype in sorghum. From an ethyl methane sulfonate (EMS)-induced Targeting Induced Local Lesions in Genomes (TILLING) population (Xin *et al.* 2009; Xin *et al.* 2008), 10 new *bmr* lines were identified in the initial study, and two were shown to have novel mutations in *Bmr12* (*COMT* gene). These other mutants may represent novel loci whose gene products are involved in lignin biosynthesis or in the regulation of this metabolic pathway. To identify new *brown midrib* lines with mutations in genes other than *Bmr2*, *Bmr6*, and *Bmr12*, a series of putative *brown midrib* mutant lines isolated from TILLING populations was subjected to allelism testing and subsequent agronomic and chemical characterization. The objectives of these studies were to identify and describe novel *brown midrib* loci and additional alleles of *bmr2*, *bmr6*, and *bmr12*.

## Materials and Methods

### Allelism tests

Forty-six putative *bmr* mutants were isolated from the M_2_ generation of an EMS mutagenized TILLING population of BTx623 (Xin *et al.* 2009; Xin *et al.* 2008), which was derived from approximately 3000 M_1_ lines. M_3_ plants shown in Table 1 were crossed to the tester lines A-MP11 (*bmr*2; USDA-ARS GRIN PI 602898; http://www.ars-grin.gov/), A-N603 (ATx623 *bmr6*; PI 639713) (Pedersen *et al.* 2006), and A-N604 (ATx623 *bmr*12; PI639714) (Pedersen *et al.* 2006) in the greenhouse at the University of Nebraska–Lincoln during winter 2008–2009. Twelve seeds for each test-cross, mutant line, and check line were planted in the greenhouse in spring 2009; including these check lines not indicated above (A/BMP12, *bmr2*, PI 602900/602901; A/BMP16, *bmr6*, PI 602908/602909; A/BMP17, *bmr6*, PI 602910/602911; A/BMP454, *bmr6* and *bmr12*, PI 602739/602740) (Pedersen *et al.* 2006). The plants were visually classified as being *brown midrib* (*bmr*) or wild-type (WT) phenotype when the plants were approximately 0.5 m in height. Additional test-crosses were performed and evaluated for the complementation tests that yielded inconclusive results in the 2009 evaluation. Digital images were collected to document the leaf midrib phenotype for test-crosses and mutant lines.

**Table 1:** Midrib genotypes of *bmr* mutant lines based on results of test-crosses with *bmr2*, *bmr6*, and *bmr12* tester lines

		Tester Line			
		AOK11 *bmr2*	AN603 (ATx623 *bmr6*)	AN604 (ATx623 *bmr12*)	
Line	Mutant Phenotype		Progeny Phenotype		Mutant Locus
23	*bmr*	WT	*bmr*	WT	*bmr6-23*
29	*bmr*	WT	WT	WT	New *bmr*
30	*bmr*	WT	WT	*bmr*	*bmr12-30*
31	*bmr*	WT	*bmr*	WT	*bmr6-31*
32	*bmr*	WT	*bmr*	WT	*bmr6-32*
34	*bmr*	WT	WT	*bmr*	*bmr12-34*
35	*bmr*	WT	WT	*bmr*	*bmr12-35*
41	*bmr*	WT	WT	WEAK	Inconclusive
45	*bmr*	WT	*bmr*	WT	*bmr6-45*
100	*bmr*	WT	WT	WT	New *bmr*
122	*bmr*	WT	WT	WT	New *bmr*
307	*bmr*	WT	*bmr*	WT	*bmr6-307*
562	*bmr*	*bmr*	WT	WT	*bmr2-2*
741	*bmr*	WT	*bmr*	WT	*bmr6-741*
820	*bmr*	WT	WT	*bmr*	*bmr12-820*
917	*bmr*	WT	*bmr*	WT	*bmr6-971*
1103	*bmr*	WT	*bmr*	WT	*bmr6-1103*
1107	*bmr*	WT	WT	WT	New *bmr*
1168	*bmr*	WT	WT	WT	New *bmr*
1277	*bmr*	WT	*bmr*	WT	*bmr6-1277*
1937	*bmr*	WT	WT	WT	New *bmr*
Tester lines					
BN603	*bmr*	WT	*bmr*	WT	*bmr6*
BN604	*bmr*	WT	WT	*bmr*	*bmr12*
BTx623^T^	WT	WT	WT	WT	
MP11 (PI 602899)	*bmr*	*bmr*	WT	WT	*bmr2*
MP12 (PI 602901)	*bmr*	*bmr*	WT	WT	*bmr2*
MP16 (PI 602909)	*bmr*	WT	*bmr*	WT	*bmr6*
MP17 (PI 602911)	*bmr*	WT	*bmr*	WT	*bmr6*
MP454 (PI 602740)	*bmr*	WT	*bmr*	*bmr*	*bmr6* and *bmr12*

The F_1_ individuals, mutant lines, and check lines were visually classified as being *brown midrib* (*bmr*) or wild-type (WT) phenotype (see *Materials and Methods*). Based on the test-crosses, loci and alleles were designated and appear in the far right column. *bmr2-2*, *bmr12-30*, *bmr12-34*, *bmr12-35*, and *bmr12-820* alleles identified in this study were characterized and reported previously (Saballos *et al.* 2012; Sattler *et al.* 2012)

Lines that were confirmed to be *brown midrib* mutants and produced WT test-cross progeny with all three tester lines were selected as having *brown midrib* alleles not allelic to *bmr2*, *bmr6*, or *bmr12*. This subset of mutant lines was crossed in all possible combinations using hand emasculation to facilitate crossing in the greenhouse during winter 2009–2010. Progeny were classified by phenotype as described above in the greenhouse during winter 2010–2011.

### Agronomic and chemical evaluation of mutants

M_3_-generation seed received from USDA-ARS in Lubbock, Texas, was increased through self pollinations under pollination bags in the greenhouse during winter 2008–2009 to produce adequate M_4_-generation seed to plant field studies at the University of Nebraska Agricultural Research and Development Center near Ithaca, Nebraska **(**Sharpsburg silty clay loam; fine, smectitic, mesic Typic Argiudoll) in 2009 and 2010. Checks BTx623^T^ (line ARS Lubbock, Texas; EMS mutagenized to generate the *bmr* mutants), BTx623^N^ (line maintained by ARS Lincoln, NE), RTx430, BWheatland, N603, N604, and BMP11 were included in these field studies for comparison. Nitrogen fertilizer was applied at 157 kg ha^−1^ prior to planting. The experimental design was a randomized complete block with four replications in each year. Plots consisted of 7.6-m rows spaced 0.76 m apart. Single-row plots of each line were planted 21 May 2009 and 25 May 2010. Atrazine [6-chloro-*N*-ethyl-*N*′-(1-methylethyl)-1,3,5-triazine-2,4-diamine] was applied at 2.2 kg ha^−1^ immediately after planting, followed by an application of quinclorac (3,7-dichloro-8-quinolinecarboxylic acid) and atrazine at 0.37 kg ha^−1^ and 1.1 kg ha^−1^, respectively, approximately 14 days after emergence. Supplemental irrigation (2.5 cm) was applied on 6 August 2009, 29 August 2009, and 9 August 2010. For plants in each plot, time to 50% anthesis was recorded and height was measured to the top of the mature panicle immediately prior to harvest. Single-plant grain-free forage samples for laboratory analyses were collected by randomly removing three plants from each plot (total of 12 plants per entry), discarding the panicle, and coarsely grinding individual stalks using a commercial silage cutter modified for small plot use (Pedersen and Moore 1995). These individual plant samples were dried in forced-air ovens at 42°, and then stored for subsequent analyses. Total biomass yield was determined by harvesting the remainder of each plot using the same modified commercial silage cutter. Sub-samples were collected and dried in a forced-air oven at 60° to determine dry matter for calculation of plot dry matter yields. Stand counts (number of plants per plot) were made immediately prior to harvest. Plots were harvested 7 October 2009 and 4 October 2010.

The single-plant forage samples were prepared for near-infrared reflectance spectrometry (NIRS) and chemical analyses by grinding in a Wiley mill (2-mm screen; Arthur H. Thomas Co., Philadelphia, PA), followed by grinding to pass a 1-mm screen on a cyclone mill (Udy Corp., Fort Collins, CO). All samples were scanned on a Model 6500 near-infrared reflectance spectrometer (NIRS Systems, Silver Spring, MD^3^). To cover the expected wide range in spectral diversity, 133 reference samples were selected using cluster analysis of the reflectance data for wet chemistry analysis (Shenk and Westerhaus 1991). Standard wet chemistry methods were used to determine crude protein (CP) (Miller *et al.* 1998), neutral detergent fiber (NDF), acid detergent fiber (ADF), and acid detergent lignin (ADL) concentration using an ANKOM 200 fiber analyzer of reference samples (ANKOM Tech. Corp., Fairport, NY) (Vogel *et al.* 1999). Total carbon and ash of reference samples were determined by Ward Labs (Kearney, NE) (Padmore 1990; Leco 2005). The wet chemistry values were then used to develop NIRS prediction equations by partial least squares (Shenk and Westerhaus 1991) and to generate observed values for each plot in each year. The calibration statistics for each trait are shown in Supporting Information, Table S1.

### Klason lignin concentration, enzymatic glucose yields, and pyrolysis of new mutant lines

#### Klason lignin analysis:

A subset of the ground single-plant samples described above containing mutants identified as being non-allelic with *bmr2*, *bmr6*, and *bmr12* was selected for determination of Klason lignin, and the samples from 2009 were analyzed. Samples were extracted in warm (60°) 50% (v/v) ethanol for 45 min to remove soluble sugars, minerals, and unbound phenolics and oven-dried at 50°. One hundred milligrams of extractive-free sorghum stover was used to determine the Klason lignin concentration. Klason lignin is defined as the ash-corrected residue remaining after the cell wall polysaccharides have been removed via a two-stage hydrolysis in concentrated (12 M) and dilute (0.4 M) sulfuric acid. The procedure was based on the method described Theander and Westerlund (1986), with the modifications of Hatfield *et al.* (1994).

#### Enzymatic saccharification:

A volume of 9.7 mL enzyme cocktail containing a 1:1 mix of *Trichoderma reesei* cellulase and Novozyme 188 (both from SigmaAldrich, St. Louis, MO) in 50 mM sodium citrate buffer pH 4.8 was added to 300 mg of extractive-free stover and incubated at 50° in a shaker-incubator. Novozyme 188 is a β-glucosidase (cellobiase) enzyme preparation from *Aspergillus niger* that results in the hydrolysis of cellobiose to glucose. The enzyme cocktail had a cellulase activity of 60 FPU/g cellulose based on NREL standard activity measurement LAP006 (Adney and Baker 1996), and the cellobiase activity in the cocktail was 2.0 CBU/mL (based on information from Sigma-Aldrich). The cocktail also contained tetracycline at a final concentration of 20 μg/mL to prevent microbial growth. Hydrolysate samples (100 μL) were collected at 4, 20, and 96 hr, placed in boiling water to inactivate the enzymes, cooled, centrifuged, and used to determine the glucose concentration using a calibrated OneTouch UltraSmart blood glucose meter (Roche Diagnostics, Indianapolis, IN) according to the procedure described by Vermerris *et al.* (2007). Samples from a minimum of six plants per entry were analyzed, and this analysis included two to three technical replicates per sample for the experiment described above.

#### Pyrolysis-GC-MS:

Stover samples (∼0.2 mg) from WT and *bmr* lines were pyrolyzed in a Varian 1079 programmable temperature vaporization (PTV) injector mounted on a Varian 3800 gas chromatograph connected to a Varian 1200 mass spectrometer. The PTV injector was operated at a split ratio of 1:100 at 450°. The pyrolysate was led on a capillary column (25 m, 0.32 mm i.d., fused silica coated with SGE BPX5). Helium (1.2 ml/min) was used as the carrier gas. The GC program started with a hold at 70° for 4 min, followed by a temperature increase to 150° at a rate of 2°/min. The temperature was subsequently increased to 250° at a rate of 4°/min, then to 320° at a rate of 30°/min. The mass spectrometer was operated at 1.0 kV. The mass range included *m*/*z* 45 to 350 and was scanned every 0.2 sec. The identification of compounds was based on Ralph and Hatfield (1991). To compare the proportion of the hydroxycinnamic acids *p*-coumaric acid and ferulic acid in the biomass, stover was subjected to pyrolysis-TMAH-GC-MS as described by Saballos *et al.* (2012). Samples of approximately 0.2 mg stover from BTx623 and the novel *bmr* mutants were *trans*-methylated with 2.5% (v/v) tetramethyl ammonium hydroxide (TMAH; Sigma-Aldrich, St. Louis, MO) in methanol (Mulder *et al.* 1992) and pyrolyzed in a Varian 1079 PTV injector mounted on a Varian 3800/1200 GC-MS as described above.

### Statistical analysis

The data were analyzed using the PROC MIXED procedure of SAS/STAT software^4^ (SAS, 2002–2008). For analyses of agronomic and chemical evaluations, years and replications were considered random effects (variables). Least squares (LS) means for mutant and check lines were generated and compared using the ADJUST = SIMULATE option in the LSMEANS/DIFF statement to control type I error and to permit multiple comparisons without the requirement for F-test significance (Littell *et al.* 2006). The rejection level for tests of significance among means was set at *P* ≤ 0.20 as recommended by FAO (Annicchiarico 2002) to control and achieve a better balance between type I and type II error rates for such experiments (variety trials with large numbers of entries). For analyses of yield data, stand count was treated as a covariate and LS means were adjusted for stand. For analyses of laboratory traits, values from the three individual plant samples per plot were considered repeated measures. Means of all variables were ranked from highest to lowest, and means not significantly different from BTx623^T^ were identified. For Klason lignin and enzymatic saccharification analyses, LS means for mutant and check lines were generated and compared using the LSMEANS/DIFF statement, and significance level for tests among means was set at *P* ≤ 0.05 because of the smaller number of entries.

## Results

### Determining allelism among the *bmr* mutants

**Figure 2:**
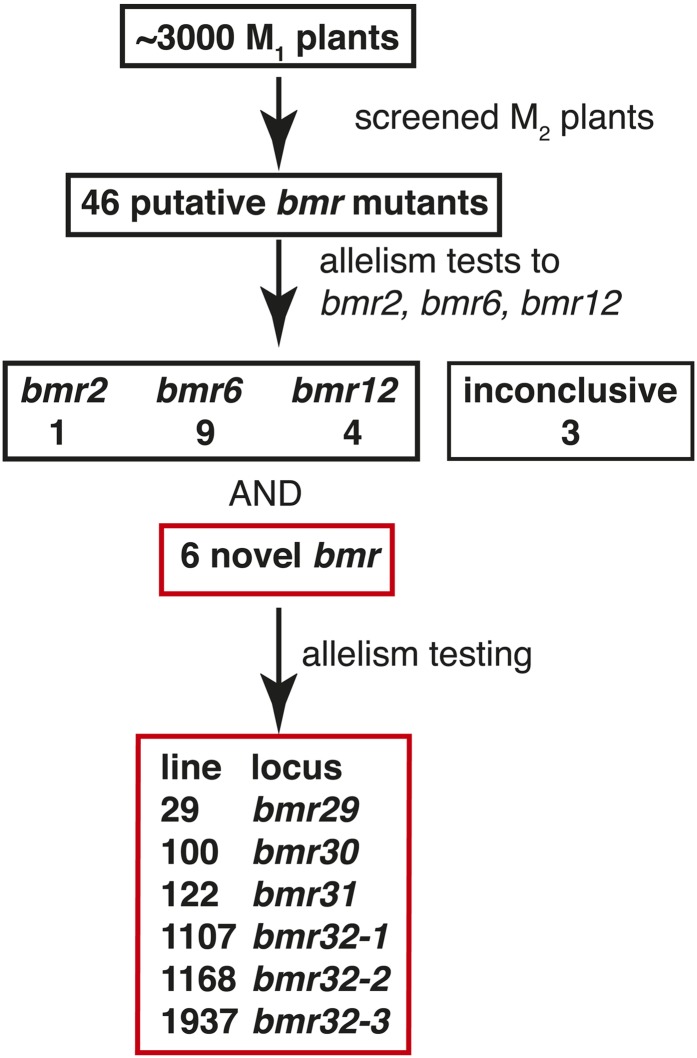
This diagram summarizes the results of the screening process that identified the *bmr* mutants in the three characterized loci and the four newly identified loci.

**Figure 3:**
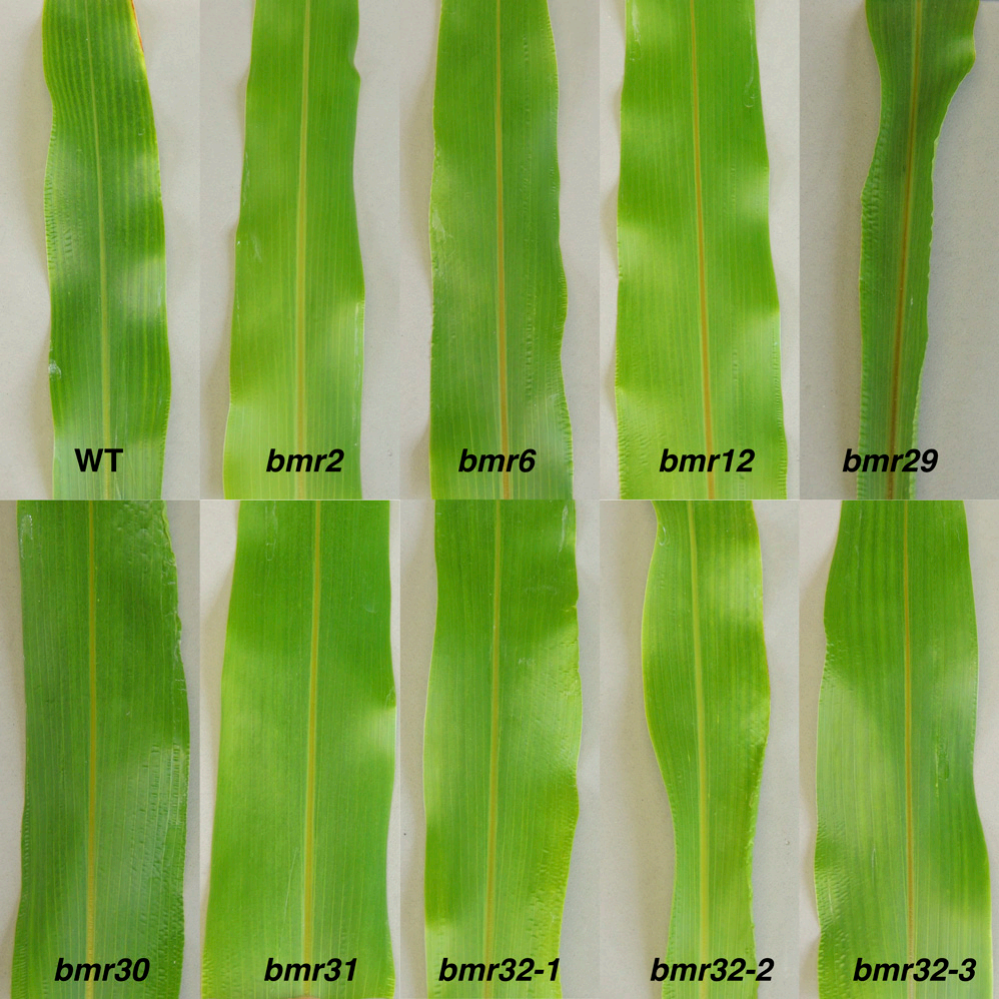
The leaf midrib phenotype of the wild-type BTx623^T^ (WT), B OK11 *bmr2*, BTx623 *bmr6*, BTx623 *bmr12*, *bmr29*, *bmr30*, *bmr31*, *bmr32-1*, *bmr32-2*, and *bmr32-3*. The sixth leaf was photographed from 6-wk-old plants.

In the greenhouse, putative *bmr* mutant lines were crossed to cytoplasmic male-sterile lines representing the three previously described *bmr* loci, *bmr2*, *bmr6*, and *bmr12*, and the leaf midrib phenotype of F1 progeny and mutant lines were visually scored when the plants were approximately 0.5 m tall (Table 1). Through this visual examination, the midribs of 23 of the lines were determined to fall within the naturally observed range of midrib color, and to not have a *brown midrib* phenotype (Table S2). For three lines, the results of these test-crosses were inconclusive (Table S2). In addition, one allele of *bmr2*, nine alleles of *bmr6*, and four alleles of *bmr12* were identified based on leaf phenotype of the F1 progeny (Figure 2 and Table 1). The line number was incorporated into the allelic designation for these previously described *bmr* loci—*bmr2*, *bmr6*, or *bmr12*. The mutations responsible for the *bmr2-2*, *bmr12-30*, *bmr12-34*, *bmr12-35*, and *bmr12-820* alleles isolated were characterized and reported previously (Saballos *et al.* 2012; Sattler *et al.* 2012). In addition, there were six mutant lines with *bmr* leaf phenotypes that were not allelic to *bmr2*, *bmr6*, or *bmr12*. The leaf midrib phenotypes of these six mutant lines were similar to the previously described loci *bmr2*, *bmr6*, or *bmr12*, although brown coloration was not dark as *bmr6* midrib (Figure 3). Additional crosses between these six lines were performed and the F1 progeny were scored for the *bmr* leaf midrib phenotype to determine allelism among them (Table 2). The results of these test-crosses indicated that the six *bmr* mutant lines (29, 100, 122, 1107, 1168, and 1937) represent four novel loci (Figure 2), which we named *bmr29* through *bmr32* to avoid overlap with the designators *bmr1* through *bmr28* for *bmr* mutants previously isolated at Purdue University in the 1970s (Porter *et al.* 1978; Saballos *et al.* 2008). Mutant line 41 was excluded from this group because its leaf phenotype and the results of the allelism tests were inconsistent (Table 2). In summary, 20 lines appear to carry mutations responsible for the *bmr* leaf midrib phenotype.

**Table 2:** Midrib phenotype of progeny from test-crosses among seven mutant lines not allelic to *bmr2*, *bmr6* or *bmr12*

♀ Parent	♂ Parent						
	29	41	100	122	1107	1168	1937
29	–	WT	WT	WT	WT	WT	WT
41	WT	–	WT	*bmr*	WT	WT	WT
100	WT	WT	–	WT	WT	WT	–
122	WT	?	WT	–	WT	WT	WT
1107	WT	WT	WT	WT	–	*bmr*	*bmr*
1168	WT	WT	WT	WT	*bmr*	–	*bmr*
1937	WT	WT	WT	WT	*bmr*	*bmr*	–

The F_1_ individuals, mutant lines, and check lines were visually classified as being *brown midrib* (*bmr*), wild-type (WT), or inconclusive (?) (see *Materials and Methods*).

### Field evaluation of *bmr* mutant lines

Concurrently, the *bmr* mutant lines were grown in the field to evaluate agronomic characteristics over two seasons (Table S3). At full maturity, biomass samples were collected for chemical analysis. Time to 50% anthesis was recorded, and the putative *bmr* mutant lines did not show statistically significant differences (analysis of lsmeans; *P* < 0.2) compared with BTx623^T^, from which these lines were derived through EMS mutagenesis (Table S3). Three lines (*bmr6-307*, *bmr30*, and *bmr32-3*) had statistically significantly reduced stand counts (plants/m) relative to BTx623^T^ (Table S3). Eight of the 20 *bmr* lines had statistically significantly reduced plant heights (m) at maturity relative to BTx623^T^. Significant reductions in dry matter yield (T/ha) of the above-ground biomass (stover) were observed for all 20 lines relative to BTx623^T^ (Table S3). Statistically significant differences in stover yield relative to BTx623^T^ were not observed for any of the control lines, including the BTx623 near-isogenic lines containing *bmr6-ref* or *bmr12-ref*.

### Analysis of stover chemical composition of *bmr* mutant lines

**Figure 4:**
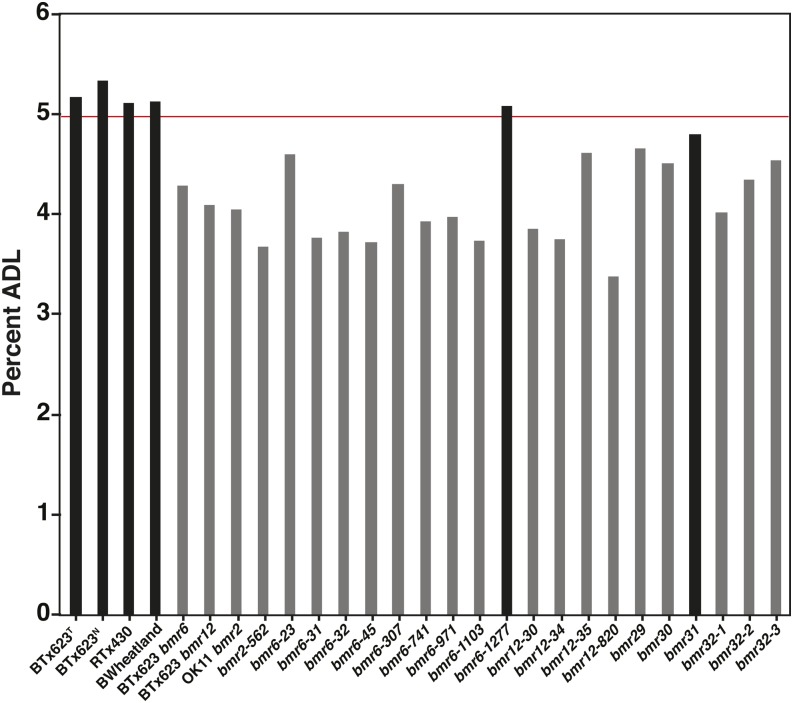
Acid detergent lignin (ADL) concentrations were determined by NIRS prediction based on the calibration equation (Table S1). Additional details are found in the *Materials and Methods* section. The values presented are least squares means (lsmean) for *bmr* mutant and check lines (Table S4). *Gray bars* indicate values that were statistically significantly different (*P* ≤ 0.20) from the values of BTx623^T^, the line used for mutagenesis. The *red line* indicates lower limit of all ranked variables for WT (BTx623^T^). Additional details of the statistical analysis are described in the *Materials and Methods* section.

The stover chemical composition was determined through NIRS to determine the impact these 20 *bmr* mutants had on lignin concentrations (Table S4). Three lines (OK11 *bmr2*, *bmr6-45*, *bmr6-1277*) had statistically significant differences for neutral detergent fiber (NDF) relative to BTx623^T^ (Table S4), which reflects the amount of cellulose, hemicellulose, and lignin in the stover. Fourteen lines showed statistically significant differences for acid detergent fiber (ADF) relative to BTx623^T^ (Table S4), which reflects the combined amount of cellulose and lignin in the stover. Twelve of the *bmr* lines had reduced ADF levels, which was likely due to reduced lignin concentration of the stover (Table S4). However, two lines (*bmr6-1277* and *bmr29*) had significantly higher ADF levels than WT, which was unexpected. Except for *bmr6-1277* and *bmr31*, all *bmr* mutant lines (Table 1) had significantly reduced acid detergent lignin (ADL) levels relative to WT BTx623^T^ (Figure 4 and Table S4). The lowest ADL level was observed in *bmr12-820* (lsmeans 3.37), which represents an approximate 35% reduction in ADL relative to BTx623^T^ (lsmeans 5.18). Hence, these results are consistent with previously reported reductions in ADL levels of the stover associated with *bmr6* and *bmr12* phenotypes (Oliver *et al.* 2005a, b; Sattler *et al.* 2010).

In addition, the total ash concentration, crude protein concentration, and total carbon concentration of the stover were determined through NIRS (Table S5). Eight lines had significantly reduced ash concentration relative to WT BTx623^T^, whereas four lines (*bmr2* and *bmr6* checks, *bmr6-45*, *bmr6-307*) had significantly increased ash concentration (Table S5). Four lines (*bmr6*-307, *bmr12-30*, *bmr31*, *bmr32-3*) showed significant increases in crude protein concentration of stover (Table S5), which has been associated with abiotic and biotic stresses (Buxton 1996; Wilkins and Humphreys 2003). Total carbon concentration of the stover was significantly increased in eight *bmr* lines. Total carbon concentration was significantly decreased for three lines, *bmr6-307*, and the *bmr2* and *bmr6* checks, all of which had significantly increased ash concentration of the stover. There were significant differences in total carbon or ash concentration of the stover for *bmr* lines confirmed through complementation tests, but these results did not follow a trend except for lines allelic to *bmr12* and *bmr32* (Table S5). Stover of all three *bmr32* lines had a significant decrease in ash concentration and a significant increase in total carbon concentration. Similarly, stover from three of the four *bmr12* lines, but not the check BTx623 *bmr12-ref*, had a significant decrease in ash concentration, and three of the four *bmr12* lines had a significant increase in total carbon concentration.

### Characterizing the chemical composition of four novel *bmr* mutants

The effect of the four novel *bmr* mutations on Klason lignin concentration was determined, and *bmr29* stover had a statistically significant reduction (of 14.3%) in Klason lignin relative to WT BTx623^T^ at *P* ≤ 0.05 (Table 3). The stover from *bmr31* and *bmr32-1* through *bmr32-3* had lsmeans for Klason lignin that were lower than lsmeans for WT stover, but at the 0.2 level rather than the 0.05 level of probability. Overall, the lower ranges of Klason lignin concentration for stover from these mutants (Table 3) combined with the ADL data (Table S4) suggest that these mutations reduce lignin concentration, but that the effect is, overall, smaller than in the *bmr2*, *bmr6*, and *bmr12* mutants. Other EMS-induced mutations in these lines may contribute to the observed variation among the samples (see *Discussion*).

**Table 3:** Klason lignin concentration and saccharification efficiency of stover from four newly identified *bmr* loci

				Glucose Release (mg/g Stover)								
	Klason Lignin (%)			Hydrolysis 4 hr			Hydrolysis 20 hr			Hydrolysis 96 hr		
Line	lsmean	Lower	Upper	lsmean	Lower	Upper	lsmean	Lower	Upper	lsmean	Lower	Upper
BTx623^T^	21.2	20.5	22.0	39.9	28.9	50.8	57.7	47.1	68.2	68.8	60.5	77.0
*bmr29*	**18.2**	**16.7**	**19.7**	39.2	30.2	48.1	49.5	38.9	60.0	57.3	49.1	65.5
*bmr30*	20.2	18.3	22.1	51.4	40.5	62.4	73.9	63.3	84.4	79.5	71.2	87.7
*bmr31*	19.7	18.1	21.2	54.7	43.8	65.7	61.1	50.6	71.7	74.3	66.1	82.5
*bmr32-1*	19.2	17.2	21.1	41.6	28.2	55.0	55.3	42.4	68.2	65.0	54.9	75.2
*bmr32-2*	ND	ND	ND	43.0	29.6	56.5	58.9	46.0	71.8	59.7	49.2	70.2
*bmr32-3*	19.2	17.5	21.0	44.5	36.4	52.6	58.0	50.2	65.8	65.5	59.1	71.8

Klason lignin concentration was determined as described in the *Materials and Methods* section. Glucose yields were obtained after enzymatic saccharification with cellulase of native (unpretreated) stover after 4, 20, and 96 hr. Least squares means (lsmean) for mutant and check lines were generated, and means of all variables were ranked from highest (Upper) to lowest (Lower). **Bold text** indicates values that were statistically significantly different (*P* ≤ 0.05) from the values of BTx623^T^, the line used for mutagenesis. Additional details of the statistical analysis are described in the *Materials and Methods* section.

To assess the potential of these novel *bmr* mutations for improving the biomass conversion efficiency of the stover, enzymatic saccharification of the cellulose was performed. Ground native (unpretreated) stover samples were treated with cellulases, and the amount of glucose released was monitored at fixed time points. The stover from two of the mutants, *bmr30* and *bmr31*, yielded more released glucose than WT at *P* ≤ 0.2 at 4 hr for *bmr30*, and at 20 hr and 96 hr for *bmr31* (Table 3). Interestingly, *bmr29* stover, which had the lowest mean Klason lignin concentration among the novel *bmr* mutants, did not release more glucose than the WT control following enzymatic saccharification (Table 3). Hence, a clear relationship between glucose release following saccharification and lignin concentration, determined as either Klason lignin or ADL, was not observed.

**Figure 5:**
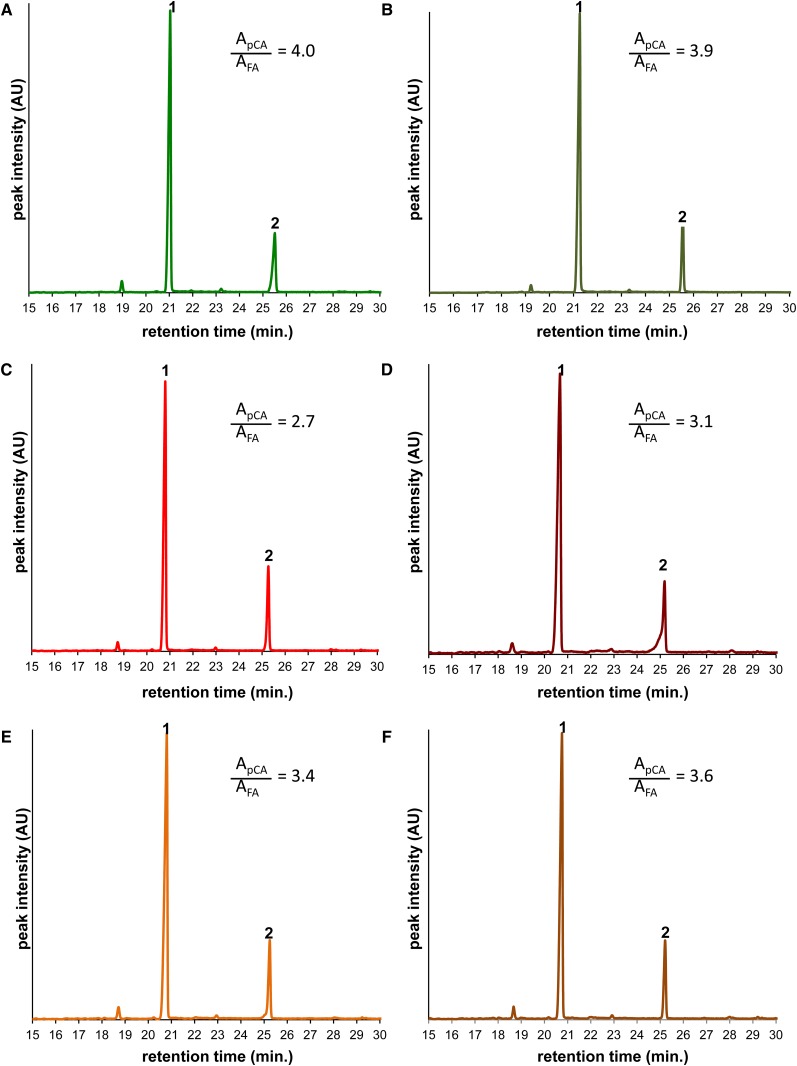
Normalized total ion current chromatograms displaying the content of esterified *p*-coumaric (pCA) and ferulic acid (FA) in the cell walls of wild-type and *bmr* stover as determined by pyrolysis-TMAH-GC-MS. (A) BTx623 (wild-type). (B) *bmr29*. (C) *bmr30*. (D) *bmr31*. (E) *bmr-32-1*. (F) *bmr32-2*. The peaks representing the double methylesters of (1) *trans-p*-coumaric acid (*m*/*z* 192) and (2) *trans*-ferulic acid (*m*/*z* 222) have been selectively displayed. The ratio of the peak areas (pCA/FA) is displayed. This ratio is not reflective of the actual molar ratio of pCA and FA in the cell wall due to the differences in sensitivity of detection.

Lignin subunit composition resulting from the novel *bmr* mutations was analyzed using pyrolysis-GC-MS. Unlike what has been observed in the *bmr2* (Saballos *et al.* 2012), *bmr6* (Saballos *et al.* 2009; Sattler *et al.* 2009), and *bmr12* mutants (Bout and Vermerris 2003; Palmer *et al.* 2008), no apparent changes in phenolic compounds derived from either guaiacyl or syringyl residues were identified (Figure S1). Stover from three of the four novel *bmr* mutants (*bmr30*, *bmr31*, *bmr32*) did, however, contain more ferulic acid based on analysis of the pyrograms obtained from TMAH-treated stover (Figure 5). Ferulic acid is primarily esterified to the hemicellulosic polysaccharide glucuronoarabinoxylan (GAX), where it serves as a nucleation site for lignification. The higher content of ferulic acid identified in the pyrograms is consistent with the higher hemicellulose content observed in the *bmr30*, *bmr31*, and *bm32* mutants, as calculated by subtracting ADF from NDF (Table S4) (27% for BTx623 *vs.* 29% for the three *bmr* mutants). The increased ferulate levels in these *bmr* mutants may reflect cell wall compositional and/or architectural changes that may influence the rate of glucose release during enzymatic saccharification from *bmr30* and *bmr31* stover.

## Discussion

In this study, 20 novel *bmr* mutant lines were identified and characterized based on allelism tests. Among this set of 20, six *bmr* mutants were not allelic to *bmr2*, *bmr6*, or *bmr12*. Further complementation testing showed these mutants represent four novel *bmr* loci. Hence, this study has doubled the number of *bmr* loci identified and characterized in sorghum from the original four loci *bmr2*, *bmr6*, *bmr12*, and *bmr19* isolated and described at Purdue University (Porter *et al.* 1978; Saballos *et al.* 2008), although it should be noted that *bmr19* was not available to test whether it is allelic to any of the six new mutants (*bmr29* through *32*). Pyrolysis-GC-MS analysis of *bmr19* stover showed a reduced amount of G-derived pyrolysis products relative to WT (Saballos *et al.* 2008), and this change was not observed in the any six novel mutants described here, which suggests that none of the *bmr* mutants described here represent alleles of *bmr19*. The *bmr* mutants obtained from this TILLING population provide both novel *bmr* sources for plant breeding and new tools to study lignin synthesis and bioenergy conversion processes. However, the relative ease in which *bmr* mutants were isolated from this mutagenized population also has an obvious drawback for their subsequent uses. Previously, it was estimated that there was an EMS-induced mutation every 516 kb within the population (Xin *et al.* 2008). These heavy mutation loads affect overall plant fitness based on evaluation of agronomic traits (Table S3). Although most lines were not statistically different from the WT line BTx623^T^ for stand count, time to 50% anthesis, and plant height, dry matter (stover) yield was significantly reduced in all 20 *bmr* lines. In contrast, the near-isogenic *bmr6* and *bmr12* lines previously introgressed into BTx623 (Pedersen *et al.* 2006) had stover yields that were not significantly different from WT (Table S3). Clearly, these new *bmr* lines will require several generations of backcrossing to reduce the number of deleterious mutations each line is carrying before they can be used in plant breeding programs.

The *brown midrib* mutations have been previously shown to reduce the lignin concentration of biomass in maize, sorghum, and pearl millet (*Pennisetum glaucum* L.) (Kuc and Nelson 1964; Gee *et al.* 1968; Porter *et al.* 1978; Cherney *et al.* 1988). Likewise, stover from all *bmr* lines had significantly reduced ADL levels except for *bmr6-1277* and *bmr31*, which were not significantly different from WT (Figure 4). The ash and total carbon concentration of the stover were significantly different than the WT BTx623^T^ for some of the *bmr* lines (Table S5). However, there were also significant differences for these two traits between BTx623^T^ stover and the stover from four of the control lines (RTx430, BTx623 *bmr6*, OK11 *bmr2*). These results may indicate that both ash and total carbon concentration are more variable components of stover as compared with NDF, ADF, and ADL. The inverse relationship between significant differences in ash concentration and total carbon concentration that was observed for nine of the *bmr* stover samples may reflect the influence of ash concentration on measurement of total carbon concentration.

One of the goals of these experiments was to identify novel *bmr* loci and to determine whether the impact these mutations have on lignin biosynthesis translated into increased saccharification of the stover. Despite the commonly accepted view that lignin impedes enzymatic saccharification (Chen and Dixon 2007; Grabber 2005), the data presented here do not indicate a correlation between Klason lignin or ADL concentration and glucose yield following enzymatic saccharification (Table 3 and Table S4). For example, among the mutants examined, the *bmr30* mutant yields the highest amount of glucose while its Klason lignin concentration is not different from WT, and the *bmr29* mutant has the lowest amount of Klason lignin but does not yield more glucose. Prior studies have indicated that lignin subunit composition influences glucose release. In the *bmr6* and *bmr12* mutants of sorghum, the yield of fermentable sugars was shown to be not only higher (Dien *et al.* 2009; Saballos *et al.* 2008) but also additive in nature when both mutations were combined (Dien *et al.* 2009). In both of these mutants, the S/G ratio was lower (Bout and Vermerris 2003; Palmer *et al.* 2008; Sattler *et al.* 2009), largely due to the reduction in S-residues, resulting in an almost complete lack of these residues in the double mutant. Similar observations have been made in maize mutants in which the *brown midrib1* (*bm1*) and *bm3* mutations were combined (Vermerris *et al.* 2007). Other factors that have been shown to play a role in biomass conversion efficiency in maize and sorghum are the crystallinity index of the cellulose (Vandenbrink *et al.* 2012) and the concentration of hemicellulose in the stover (Torres *et al.* 2014). The latter factor may contribute to the enhanced release of glucose from the stover of *bmr30* and *bmr31*. Finally, given that the novel *bmr* mutants were identified in a TILLING population, it is possible that there are other mutations with a negative impact on enzymatic saccharification. However, it is unlikely that this would have occurred in all six of the novel *bmr* mutants.

In conclusion, the newly identified *bmr* alleles of *bmr2*, *bmr6*, and *bmr12* and four novel *bmr* loci represent tools to manipulate lignin concentration and biomass conversion. The four novel *bmr* loci may represent genes involved in lignin biosynthesis and could be used to further our understanding of this pathway in sorghum, even though they did not significantly increase saccharification yield compared with WT. Because the *bmr2*, *bmr6*, and *bmr12* mutations significantly decreased lignin concentration and increased enzymatic saccharification yields relative to WT (Bout and Vermerris 2003; Palmer *et al.* 2008; Saballos *et al.* 2008, 2012), the most practical use of the novel *bmr* mutants may be to combine them with the three previously described mutants or with each other, similar to “stacking” *bmr6* and *bmr12*, which resulted in a greater reduction in lignin concentration and larger increase in biomass conversion efficiency than achieved with either mutation alone (Sattler *et al.* 2010; Dien *et al.* 2009).

## Supplementary Material

Supporting Information
