# Supplementary material for: Characterization of Novel Sorghum brown midrib Mutants from an EMS-Mutagenized Population
Source: G3 (Bethesda). 2014 Sep 2;4(11):2115–24. doi: 10.1534/g3.114.014001 (PMC4232537; doi:10.1534/g3.114.014001)
Supplement: Supporting Information [file supp_4_11_2115__index.html]

Characterization of Novel Sorghum brown midrib Mutants from an EMS-Mutagenized Population — Supporting Information 

# Characterization of Novel Sorghum *brown midrib* Mutants from an EMS-Mutagenized Population

## Supporting Information for Sattler *et al.*, 2014

**Files in this Data Supplement:**

- Supporting Information - Tables S1-S5 and Figure S1 (PDF, 204 KB)
- Table S1 - Calibration statistics for NIRS prediction of CP, NDF, ADF, ADL, Ash, and Total Carbon. (PDF, 66 KB)
- Table S2 - Midrib phenotype and genotypes of 26 non-*bmr* lines based on results of test crosses with *bmr2, bmr6,* and *bmr12* tester lines. (PDF, 66 KB)
- Table S3 - Evaluation of the agronomic traits for the mutant lines. (PDF, 75 KB)
- Table S4 - NIRS prediction for NDF, ADF and ADL of the mutant lines. (PDF, 70 KB)
- Table S5 - NIRS prediction for Total Ash, Crude Protein and Total Carbon of the *bmr* mutant lines. (PDF, 70 KB)
- Figure S1 - Partial pyrograms obtained with stover from A. BTx623, B. *bmr30*, C. *bmr31*, D. *bmr32-1*, E. BTx623, and F. *bmr29*. (PDF, 136 KB)
