# Supplementary material for: Characterization of Novel Sorghum brown midrib Mutants from an EMS-Mutagenized Population
Source: G3 (Bethesda). 2014 Sep 2;4(11):2115–24. doi: 10.1534/g3.114.014001 (PMC4232537; doi:10.1534/g3.114.014001)
Supplement: Supporting Information [file supp_g3.114.014001_014001SI.pdf]

G3·Genes | Genomes | Genetics

Characterization of novel sorghum *brown midrib* mutants from an EMS-mutagenized population

Scott E. Sattler[*,§], Ana Saballos[**,1], Zhanguo Xin[§§], Deanna L. Funnell-Harris[*,¶], Wilfred Vermerris[€] and Jeffrey F. Pedersen[*,§]

[*]Grain, Forage and Bioenergy Research Unit, USDA-ARS, Lincoln, NE 68583

[§]Department of Agronomy and Horticulture, University of Nebraska–Lincoln, Lincoln, NE 68583

[**]Agronomy Department and Genetics Institute, University of Florida, Gainesville, FL 32610

[§§]Plant Stress and Germplasm Development Unit, USDA-ARS, Lubbock, TX 79415

[¶]Department of Plant Pathology, University of Nebraska–Lincoln, Lincoln, NE 68583

[€]Department of Microbiology & Cell Science and Genetics Institute, University of Florida, Gainesville, FL 32610

[1]Present Address: Chromatin Inc., Alachua, FL 32615

[2]The U.S. Department of Agriculture (USDA) prohibits discrimination in all its programs and activities on the basis of race, color, national origin, age, disability, and where applicable, sex, marital status, familial status, parental status, religion, sexual orientation, genetic information, political beliefs, reprisal, or because all or part of an individual's income is derived from any public assistance program. (Not all prohibited bases apply to all programs.) Persons with disabilities who require alternative means for communication of program information (Braille, large print, audiotape, etc.) should contact USDA's TARGET Center at (202) 720-2600 (voice and TDD). To file a complaint of discrimination, write to USDA, Director, Office of Civil Rights, 1400 Independence Avenue, S.W., Washington, D.C. 20250-9410, or call (800) 795-3272 (voice) or (202) 720-6382 (TDD). USDA is an equal opportunity provider and employer.

Corresponding Author: Scott Sattler, Grain, Forage and Bioenergy Research Unit USDA-ARS, 137 Keim Hall, East Campus UNL, Lincoln, NE 68583-0937; phone: 402-472-5987; FAX: 402-472-4020; e-mail: Scott.Sattler@ars.usda.gov

DOI: 10.1534/g3.114.014001

Table S1. Calibration statistics for NIRS† prediction of CP, NDF, ADF, ADL, Ash, and Total Carbon.

Trait	Math treatment‡	Calibration R^2	SEC	SECV	SEP	Validation R^2
			---------------g kg^{-1}--------------			
CP	2,8,6,1	0.989	0.228	0.303	0.517	0.982
NDF	4,10,10,1	0.939	1.371	1.563	2.231	0.882
ADF	3,10,10,1	0.955	0.852	1.107	1.164	0.931
ADL	3,5,5,1	0.882	0.373	0.606	0.577	0.805
Ash	3,10,10,1	0.963	0.476	0.662	0.449	0.937
Total Carbon	3,5,5,1	0.918	0.277	0.517	0.192	0.922

†Abbreviations: NIRS=near-infrared spectroscopy, CP=crude protein, NDF=neutral detergent fiber, ADF=acid detergent fiber, ADL=acid detergent lignin, SEC=standard error calibration, SECV=standard error cross validation, SEP=Standard Error of Prediction of validation set.
‡Math treatments used by NIRS software = derivative number, gap (nm over which derivative is calculated), smooth (number of points over which data is smoothed), second smooth (number of points).

Table S2. Midrib phenotype and genotypes of 26 non-*bmr* lines based on results of test crosses with *bmr2*, *bmr6*, and *bmr12* tester lines.

Line	Mutant Phenotype	Tester Line AOK11 *bmr2*	AN603 ATx623 *bmr6*	AN604 ATx623 *bmr12*	Mutant locus
4	WT	WT	WT	WT	
25	WT	WT	WT	WT	
39	WT	-	WT	WT	
40	WT	WT	WT	WT	
41	*bmr*	WT	WT	WEAK	inconclusive
163	WT	WT	WT	WT	
247	WT	WT	WT	WT	
371	WT	WT	WT	WT	
372	WT	WT	WT	WT	
485	WT	WT	WT	WT	
492	WT	WT	WT	WT	
557	WT	WT	WT	WT	
666	WEAK	WT	WT	WT	inconclusive
706	WT	WT	WEAK	WT	inconclusive
924	WT	WT	WT	WT	
934	WT	WT	WT	WT	
1057	WT	WT	WT	WT	
1074	WT	WT	WT	WT	
1402	WT	WT	WT	WT	
1492	WT	WT	WT	WT	
1593	WT	WT	WT	WT	
1605	WT	WT	WT	WT	
1614	WT	WT	WT	WT	
1634	WT	WT	WT	WT	
1668	WT	WT	WT	WT	
1827	WT	WT	WT	WT	

The F_1 individuals, mutant lines, and check lines were visually classified as being *brown midrib* (*bmr*) or wild-type (WT) phenotype (See Materials and Methods). Based upon the test-crosses, loci and alleles were designated, which appear in the far right column.

Table S3. Evaluation of the agronomic traits for the mutant lines

	Stand Count (plants/m)			Time to 50% Anthesis (days after planting)			Height (cm)			Yield (T/ha)		
Line	lsmean	Lower	Upper	lsmean	Lower	Upper	lsmean	Lower	Upper	lsmean	Lower	Upper
BTx623[T]	14.13	10.31	17.94	73.38	52.66	94.09	153.13	135.79	170.46	17.50	16.07	18.94
BTx623[N]	16.63	12.95	20.30	72.75	52.28	93.22	148.13	128.63	167.62	17.26	14.19	20.34
RTx430	13.13	9.49	16.76	75.75	55.17	96.34	123.75	103.13	144.37	16.63	14.83	18.44
BWheatland	14.75	10.51	18.99	73.88	53.38	94.37	**110.63**	**90.74**	**130.51**	13.71	12.34	15.08
BTx623 *bmr6*	17.38	13.07	21.68	74.00	53.31	94.69	125.00	105.47	144.53	13.66	10.59	16.72
BTx623 *bmr12*	15.75	12.03	19.47	76.63	56.57	96.68	131.88	111.06	152.69	15.72	14.09	17.35
OK11 *bmr2*	15.13	11.48	18.77	78.00	57.39	98.61	120.00	101.98	138.02	**12.02**	**10.09**	**13.94**
bmr2-2	10.00	6.26	13.74	78.88	58.83	98.92	128.13	108.97	147.28	**7.12**	**6.15**	**8.09**
bmr6-23	15.13	11.24	19.01	78.50	58.65	98.35	**96.25**	**79.52**	**112.98**	**5.23**	**4.25**	**6.21**
bmr6-31	10.50	6.85	14.15	77.50	57.40	97.60	**108.13**	**91.65**	**124.60**	**5.20**	**4.23**	**6.17**
bmr6-32	12.88	9.25	16.50	78.00	57.60	98.40	115.63	94.34	136.91	**9.76**	**8.68**	**10.83**
bmr6-45	10.63	7.01	14.24	78.13	57.73	98.52	**114.38**	**94.17**	**134.58**	**6.17**	**5.16**	**7.18**
bmr6-307	**6.25**	**2.47**	**10.03**	81.63	61.29	101.96	**112.50**	**94.06**	**130.94**	**3.48**	**1.99**	**4.96**
bmr6-741	16.50	12.86	20.14	79.50	59.11	99.89	128.13	108.96	147.29	**10.53**	**9.11**	**11.96**
bmr6-971	14.88	11.02	18.73	79.00	58.69	99.31	125.00	105.80	144.20	**11.72**	**10.47**	**12.98**
bmr6-1103	13.13	9.36	16.89	79.13	59.06	99.19	**91.88**	**71.92**	**111.83**	**8.26**	**6.88**	**9.64**
bmr6-1277	10.75	6.40	15.10	76.88	56.53	97.22	**104.38**	**83.20**	**125.55**	**7.95**	**6.66**	**9.24**
bmr12-30	8.50	4.83	12.17	86.63	66.48	106.77	118.75	101.52	135.98	**6.51**	**5.48**	**7.54**
bmr12-34	12.88	8.96	16.79	82.83	62.43	103.23	133.13	115.58	150.67	**8.45**	**7.40**	**9.49**
bmr12-35	16.25	12.48	20.02	74.13	53.57	94.68	132.50	112.12	152.88	**12.95**	**11.51**	**14.38**
bmr12-820	11.21	7.49	14.93	89.37	71.06	107.67	126.83	107.39	146.26	**4.83**	**3.53**	**6.13**
bmr29	12.25	8.33	16.17	75.50	54.86	96.14	133.13	116.21	150.04	**7.30**	**6.34**	**8.27**
bmr30	**5.25**	**0.57**	**9.93**	84.00	64.28	103.72	128.75	111.38	146.12	**4.77**	**3.53**	**6.00**
bmr31	9.21	5.22	13.20	84.87	65.34	104.39	**69.33**	**52.75**	**85.91**	**2.30**	**-0.43**	**5.03**
bmr32-1	7.88	3.84	11.91	82.25	62.52	101.98	126.88	109.42	144.33	**6.91**	**4.97**	**8.86**
bmr32-2	11.88	8.15	15.60	76.13	55.43	96.82	**115.00**	**97.51**	**132.49**	**8.26**	**7.26**	**9.27**
bmr32-3	**6.25**	**2.46**	**10.04**	82.75	63.49	102.01	117.50	97.39	137.61	**3.49**	**1.69**	**5.29**
4	12.25	8.61	15.89	78.13	57.62	98.63	125.63	106.95	144.30	**9.99**	**8.89**	**11.09**
25	11.88	8.23	15.52	87.40	69.54	105.26	148.13	131.07	165.18	**9.75**	**7.53**	**11.96**
39	8.00	4.05	11.95	84.50	63.84	105.16	**100.63**	**82.12**	**119.13**	**6.63**	**5.34**	**7.92**
40	8.63	4.92	12.33	78.75	58.25	99.25	126.88	106.25	147.50	**10.62**	**9.55**	**11.69**
41	13.75	10.03	17.47	75.50	55.20	95.80	116.88	97.06	136.69	**9.71**	**8.71**	**10.70**
163	17.38	13.69	21.06	80.75	60.29	101.21	122.50	102.23	142.77	**8.61**	**7.63**	**9.60**
247	6.71	2.35	11.07	87.20	66.97	107.43	**83.49**	**65.20**	**101.79**	**2.13**	**1.11**	**3.14**
371	14.00	10.19	17.81	79.13	59.04	99.21	149.38	132.40	166.35	15.07	13.29	16.86
372	6.88	2.55	11.20	76.25	56.22	96.28	138.13	118.44	157.81	**12.12**	**10.71**	**13.53**
485	13.38	9.63	17.12	75.75	55.05	96.45	144.38	124.26	164.49	**11.09**	**9.89**	**12.28**
492	10.75	7.11	14.39	85.25	65.89	104.61	**107.50**	**87.33**	**127.67**	**5.89**	**4.93**	**6.84**
557	9.75	6.10	13.40	90.63	70.09	111.16	130.00	113.36	146.64	**7.92**	**6.95**	**8.90**
666	14.75	10.92	18.58	77.75	57.44	98.06	134.38	113.26	155.49	**9.48**	**7.39**	**11.57**

706	7.50	3.85	11.15	84.13	63.88	104.37	**108.75**	**89.78**	**127.72**	**4.28**	**3.10**	**5.46**
924	15.00	11.35	18.65	76.13	55.58	96.67	**99.38**	**80.46**	**118.29**	**9.19**	**7.58**	**10.80**
934	8.25	3.90	12.60	88.38	69.42	107.33	**103.13**	**82.20**	**124.05**	**2.76**	**0.99**	**4.53**
1057	10.38	6.72	14.03	75.38	55.08	95.67	119.38	99.14	139.61	**6.38**	**5.43**	**7.33**
1074	14.25	10.32	18.18	74.88	54.21	95.54	135.00	113.92	156.08	**13.39**	**11.81**	**14.97**
1402	9.75	5.81	13.69	82.38	62.62	102.13	117.50	96.89	138.11	**6.00**	**4.50**	**7.51**
1492	**4.75**	**1.06**	**8.44**	79.50	59.00	100.00	121.25	100.90	141.60	**4.45**	**1.78**	**7.11**
1593	13.50	9.01	17.99	87.71	68.09	107.32	**90.63**	**74.61**	**106.64**	**3.81**	**2.80**	**4.81**
1605	11.13	7.30	14.95	77.63	57.28	97.97	166.25	146.41	186.09	**9.83**	**8.51**	**11.15**
1614	11.00	7.18	14.82	77.13	56.64	97.61	128.75	109.01	148.49	**8.37**	**7.17**	**9.58**
1634	8.13	4.46	11.79	86.63	66.51	106.74	**99.38**	**78.94**	**119.81**	**3.79**	**2.84**	**4.73**
1668	11.13	7.51	14.74	79.38	59.43	99.32	**113.13**	**96.02**	**130.23**	**7.17**	**6.14**	**8.20**
1827	11.50	7.87	15.13	79.00	58.57	99.43	129.38	110.83	147.92	**7.08**	**5.80**	**8.37**

Measurement of these traits is as described in Materials and Methods. Yield is defined as the total above ground biomass. Least Squares Means (lsmean) for mutant and check lines were generated, and means of all variables were ranked from highest (Upper) to lowest (Lower). **Bold text** indicates values that were significantly different ($P \leq 0.20$) from the values of BTx623^{T}, the line used for mutagenesis. Additional details of the statistical analysis are described in Materials and Methods. BTx623^{N} is the line maintained by ARS in Lincoln, NE, which was included for comparison.

Table S4. NIRS prediction for NDF, ADF and ADL of the mutant lines

	NDF (%)			ADF (%)			ADL (%)		
Line	lsmean	Lower	Upper	lsmean	Lower	Upper	lsmean	Lower	Upper
BTx623[T]	60.86	58.85	62.86	34.23	33.50	34.97	5.18	4.98	5.39
BTx623[N]	61.48	59.48	63.49	34.52	33.76	35.28	5.34	5.13	5.54
RTx430	64.41	62.40	66.42	**36.41**	**35.69**	**37.13**	5.11	4.92	5.31
BWheatland	64.03	62.02	66.03	**36.04**	**35.32**	**36.76**	5.13	4.93	5.33
BTx623 *bmr6*	60.76	58.75	62.76	33.76	32.97	34.54	**4.28**	**4.07**	**4.49**
BTx623 *bmr12*	62.68	60.68	64.69	34.60	33.81	35.39	**4.09**	**3.88**	**4.31**
OK11 *bmr2*	**64.79**	**62.78**	**66.79**	35.53	34.79	36.27	**4.05**	**3.85**	**4.25**
bmr2-2	58.84	56.84	60.84	**31.96**	**31.22**	**32.70**	**3.67**	**3.47**	**3.88**
bmr6-23	60.84	58.83	62.85	32.82	32.13	33.51	**4.59**	**4.40**	**4.77**
bmr6-31	59.10	57.09	61.11	**31.19**	**30.49**	**31.89**	**3.77**	**3.58**	**3.97**
bmr6-32	61.02	59.02	63.02	33.05	32.31	33.78	**3.82**	**3.62**	**4.02**
bmr6-45	**54.98**	**52.98**	**56.99**	**29.30**	**28.60**	**30.00**	**3.72**	**3.53**	**3.91**
bmr6-307	58.15	56.14	60.15	**29.13**	**28.35**	**29.91**	**4.30**	**4.09**	**4.51**
bmr6-741	59.88	57.87	61.88	**32.49**	**31.77**	**33.20**	**3.92**	**3.73**	**4.12**
bmr6-971	60.14	58.14	62.15	**32.59**	**31.84**	**33.35**	**3.97**	**3.77**	**4.18**
bmr6-1103	59.44	57.43	61.44	**32.30**	**31.54**	**33.06**	**3.74**	**3.54**	**3.95**
bmr6-1277	**68.66**	**66.66**	**70.67**	**38.56**	**37.87**	**39.26**	5.08	4.89	5.27
bmr12-30	61.02	59.02	63.03	**32.40**	**31.64**	**33.16**	**3.85**	**3.64**	**4.05**
bmr12-34	61.52	59.52	63.53	33.25	32.47	34.03	**3.75**	**3.53**	**3.96**
bmr12-35	61.80	59.79	63.80	33.86	33.11	34.62	**4.61**	**4.41**	**4.82**
bmr12-820	58.46	56.44	60.49	**31.12**	**30.25**	**31.98**	**3.37**	**3.13**	**3.60**
bmr29	64.41	62.41	66.42	**37.32**	**36.58**	**38.06**	**4.65**	**4.45**	**4.85**
bmr30	61.29	59.28	63.30	**32.31**	**31.51**	**33.12**	**4.50**	**4.28**	**4.72**
bmr31	60.07	58.06	62.08	**31.48**	**30.67**	**32.29**	4.80	4.58	5.02
bmr32-1	62.34	60.33	64.34	33.52	32.78	34.26	**4.02**	**3.82**	**4.22**
bmr32-2	63.47	61.47	65.47	35.15	34.41	35.89	**4.34**	**4.14**	**4.54**
bmr32-3	59.89	57.89	61.90	**31.37**	**30.63**	**32.10**	**4.53**	**4.33**	**4.74**
4	64.05	62.05	66.06	**35.76**	**35.01**	**36.52**	5.51	5.30	5.71
25	56.90	54.90	58.91	**30.60**	**29.79**	**31.41**	**3.64**	**3.42**	**3.86**
39	61.21	59.20	63.22	32.82	32.01	33.62	4.99	4.77	5.21
40	62.45	60.44	64.45	34.33	33.57	35.09	5.47	5.26	5.68
41	60.85	58.84	62.85	33.14	32.38	33.90	5.09	4.89	5.30
163	62.53	60.53	64.54	34.63	33.87	35.38	**4.77**	**4.56**	**4.98**
247	61.51	59.50	63.53	32.73	31.90	33.57	5.20	4.97	5.43
371	**64.94**	**62.93**	**66.94**	**36.61**	**35.86**	**37.37**	5.41	5.21	5.62
372	60.40	58.39	62.40	**32.72**	**31.96**	**33.48**	5.00	4.79	5.20
485	59.26	57.26	61.27	32.88	32.15	33.62	**4.47**	**4.27**	**4.68**
492	58.94	56.93	60.95	**31.68**	**30.87**	**32.48**	**4.29**	**4.07**	**4.51**
557	59.61	57.61	61.62	**31.87**	**31.11**	**32.63**	4.78	4.57	4.98
666	**65.34**	**63.33**	**67.34**	**36.09**	**35.37**	**36.81**	**4.10**	**3.90**	**4.30**

706	59.88	57.87	61.88	**30.63**	**29.89**	**31.37**	5.58	5.38	5.78
924	**66.00**	**64.00**	**68.01**	**37.53**	**36.79**	**38.27**	**5.84**	**5.64**	**6.04**
934	62.82	60.81	64.82	33.45	32.73	34.16	5.07	4.87	5.26
1057	61.66	59.66	63.67	33.60	32.86	34.33	**4.71**	**4.51**	**4.91**
1074	59.80	57.80	61.81	32.92	32.16	33.68	**4.75**	**4.54**	**4.96**
1402	57.51	55.51	59.52	**30.97**	**30.21**	**31.73**	**4.23**	**4.02**	**4.44**
1492	60.98	58.97	62.99	**32.48**	**31.67**	**33.28**	4.93	4.71	5.16
1593	**55.73**	**53.72**	**57.74**	**29.59**	**28.91**	**30.27**	**4.14**	**3.95**	**4.32**
1605	57.65	55.64	59.66	**32.27**	**31.59**	**32.95**	5.02	4.84	5.21
1614	62.41	60.41	64.41	34.75	34.00	35.51	**5.72**	**5.51**	**5.92**
1634	57.59	55.59	59.59	**28.78**	**28.03**	**29.54**	4.80	4.59	5.01
1668	59.55	57.55	61.56	**31.57**	**30.79**	**32.35**	**4.70**	**4.49**	**4.91**
1827	**56.55**	**54.55**	**58.55**	**30.66**	**29.92**	**31.39**	**4.05**	**3.85**	**4.25**

Values for neutral detergent fiber (NDF), acid detergent fiber (ADF), and acid detergent lignin (ADL) concentration were determined by NIRS prediction based on the calibration equation (Table S1). Additional details are found in the Materials and Methods Section. Least Squares Means (lsmean) for mutant and check lines were generated, and means of all variables were ranked from highest (Upper) to lowest (Lower). **Bold text** indicates values that were statistically significantly different ($P \leq 0.20$) from the values of BTx623^{T}, the line used for mutagenesis. Additional details of the statistical analysis are described in the Materials and Methods section. BTx623^{N} is the line maintained by ARS in Lincoln, NE, which was included for comparison.

Table S5. NIRS prediction for Total Ash, Crude Protein and Total Carbon of the *bmr* mutant lines.

	Ash (%)			Crude Protein (%)			Total Carbon (%)		
Line	lsmean	Lower	Upper	lsmean	Lower	Upper	lsmean	Lower	Upper
BTx623^T	11.60	11.13	12.06	5.46	4.19	6.73	43.28	43.09	43.47
BTx623^N	11.72	11.25	12.20	5.76	4.50	7.02	43.29	43.09	43.48
RTx430	10.94	10.49	11.39	6.86	5.58	8.14	**44.03**	**43.84**	**44.21**
BWheatland	12.43	11.97	12.88	7.21	5.94	8.49	43.06	42.87	43.24
BTx623 *bmr6*	**14.52**	**14.03**	**15.01**	6.10	4.85	7.36	**41.85**	**41.65**	**42.05**
BTx623 *bmr12*	12.12	11.63	12.62	6.95	5.70	8.21	43.22	43.02	43.42
OK11 *bmr2*	**12.59**	**12.13**	**13.06**	6.66	5.39	7.93	**42.82**	**42.63**	**43.01**
bmr2-2	11.14	10.68	11.61	5.30	4.03	6.56	43.31	43.12	43.50
bmr6-23	12.28	11.84	12.71	7.19	5.90	8.49	43.03	42.85	43.21
bmr6-31	11.33	10.89	11.77	7.59	6.31	8.88	43.19	43.01	43.37
bmr6-32	10.85	10.38	11.31	6.98	5.71	8.25	43.63	43.45	43.82
bmr6-45	**10.59**	**10.15**	**11.03**	5.90	4.62	7.19	43.24	43.06	43.42
bmr6-307	**13.26**	**12.77**	**13.75**	**8.48**	**7.22**	**9.74**	**42.48**	**42.28**	**42.68**
bmr6-741	11.54	11.09	11.99	6.03	4.75	7.30	43.12	42.93	43.31
bmr6-971	11.91	11.43	12.39	6.32	5.06	7.58	43.00	42.80	43.19
bmr6-1103	11.66	11.19	12.14	6.21	4.95	7.47	43.04	42.85	43.24
bmr6-1277	**13.33**	**12.89**	**13.77**	7.03	5.74	8.31	43.17	42.98	43.35
bmr12-30	10.78	10.30	11.26	**8.04**	**6.78**	**9.30**	**43.71**	**43.51**	**43.90**
bmr12-34	**9.76**	**9.27**	**10.25**	6.58	5.32	7.83	**44.10**	**43.90**	**44.30**
bmr12-35	**10.26**	**9.78**	**10.74**	5.22	3.96	6.48	**43.94**	**43.75**	**44.13**
bmr12-820	**9.85**	**9.31**	**10.39**	7.57	6.32	8.81	43.68	43.46	43.89
bmr29	10.81	10.34	11.27	6.21	4.95	7.48	**43.83**	**43.64**	**44.02**
bmr30	**10.53**	**10.02**	**11.03**	7.63	6.38	8.88	**43.84**	**43.64**	**44.04**
bmr31	11.22	10.72	11.72	**9.72**	**8.47**	**10.98**	43.27	43.07	43.47
bmr32-1	**7.92**	**7.45**	**8.38**	5.22	3.95	6.50	**44.65**	**44.46**	**44.84**
bmr32-2	**9.86**	**9.39**	**10.32**	5.89	4.62	7.16	**44.09**	**43.90**	**44.28**
bmr32-3	**10.43**	**9.96**	**10.89**	**8.23**	**6.96**	**9.49**	**43.77**	**43.58**	**43.96**
4	11.50	11.02	11.98	7.20	5.94	8.46	43.47	43.28	43.67
25	**7.41**	**6.90**	**7.91**	4.97	3.72	6.22	**44.48**	**44.28**	**44.68**
39	**10.49**	**9.98**	**10.99**	7.83	6.58	9.08	**43.94**	**43.73**	**44.14**
40	12.23	11.75	12.70	6.93	5.67	8.19	43.28	43.08	43.47
41	10.80	10.32	11.27	6.11	4.86	7.37	43.47	43.27	43.66
163	**10.18**	**9.70**	**10.66**	6.03	4.77	7.29	**43.99**	**43.80**	**44.18**
247	12.14	11.62	12.66	**9.94**	**8.69**	**11.19**	43.39	43.18	43.60
371	11.12	10.64	11.59	5.00	3.74	6.26	43.59	43.40	43.78
372	11.36	10.89	11.84	6.35	5.09	7.61	43.40	43.21	43.59
485	**10.14**	**9.67**	**10.60**	4.47	3.20	5.74	43.49	43.31	43.68
492	**9.31**	**8.81**	**9.82**	6.60	5.35	7.85	**44.14**	**43.94**	**44.34**

557	10.87	10.40	11.35	7.48	6.22	8.74	43.48	43.28	43.67
666	**8.29**	**7.84**	**8.75**	4.95	3.67	6.23	**44.87**	**44.68**	**45.05**
706	11.07	10.61	11.54	7.68	6.42	8.95	43.20	43.01	43.39
924	12.18	11.72	12.65	7.27	6.00	8.54	**43.70**	**43.51**	**43.89**
934	11.70	11.25	12.15	**9.04**	**7.77**	**10.32**	43.46	43.27	43.64
1057	11.63	11.16	12.09	7.03	5.77	8.30	43.13	42.94	43.32
1074	11.24	10.77	11.72	5.90	4.64	7.16	43.20	43.01	43.39
1402	**9.65**	**9.17**	**10.13**	6.31	5.05	7.57	43.66	43.47	43.86
1492	10.67	10.16	11.17	6.96	5.71	8.21	43.63	43.43	43.84
1593	**10.31**	**9.88**	**10.74**	**9.06**	**7.76**	**10.35**	43.60	43.42	43.78
1605	**9.82**	**9.39**	**10.25**	6.13	4.83	7.42	**43.85**	**43.67**	**44.03**
1614	12.12	11.65	12.60	6.80	5.54	8.06	43.14	42.94	43.33
1634	**10.53**	**10.05**	**11.00**	7.41	6.15	8.67	43.53	43.34	43.73
1668	**10.24**	**9.75**	**10.73**	7.24	5.98	8.49	43.66	43.46	43.85
1827	**9.63**	**9.16**	**10.09**	5.14	3.87	6.41	**43.73**	**43.54**	**43.92**

Values for ash, crude protein and total carbon were determined by NIRS prediction based on the calibration equation (Table S1). Additional details are found in the Materials and Methods Section. Least Squares Means (lsmean) for mutant and check lines were generated, and means of all variables were ranked from highest (Upper) to lowest (Lower). **Bold text** indicates values that were statistically significantly different ($P \leq 0.20$) from the values of BTx623^{T}, the line used for mutagenesis. Additional details of the statistical analysis are described in the Materials and Methods section. BTx623^{N} is the line maintained by ARS in Lincoln, NE, which was included for comparison.

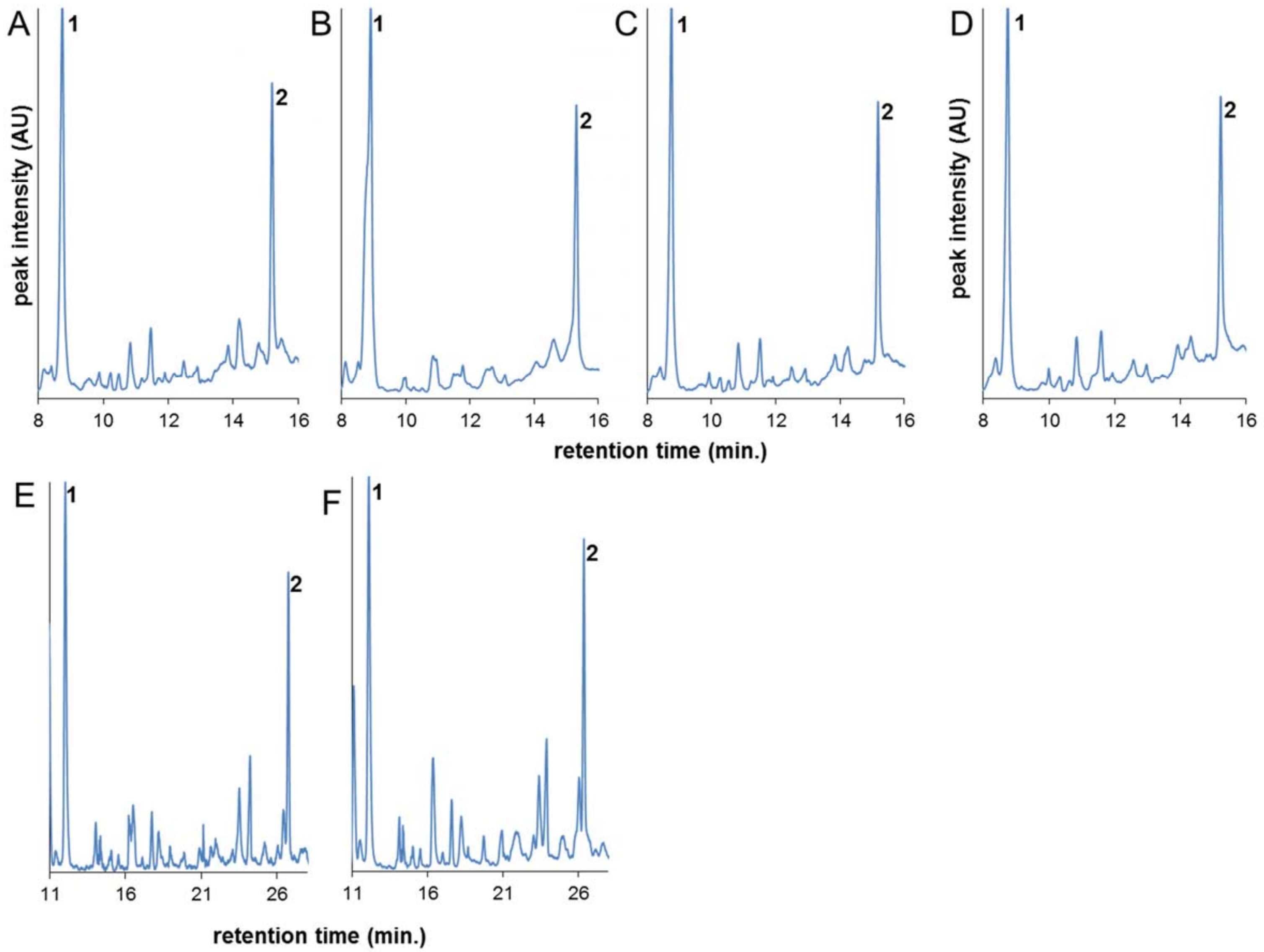

Figure S1 Partial pyrograms obtained with stover from **A.** BTx623, **B.** *bmr30*, **C.** *bmr31*, **D.** *bmr32-1*, **E.** BTx623, and **F.** *bmr29*. Peaks **1** and **2** represent 2-methoxy-4-methyl phenol (*m*/*z* 138, 123; derived from guaiacyl residues) and 2,6-dimethoxy-4-methyl phenol (*m*/*z* 168, 153; derived from syringyl residues). The difference in retention times between A-D and E, F reflects the slower ramp rate during gas chromatography of the latter two samples, to improve resolution.
