# Supplementary material for: Characterization of Novel Sorghum brown midrib Mutants from an EMS-Mutagenized Population
Source: G3 (Bethesda). 2014 Sep 2;4(11):2115–24. doi: 10.1534/g3.114.014001 (PMC4232537; doi:10.1534/g3.114.014001)
Supplement: Supporting Information [file supp_g3.114.014001_TableS1.pdf]

Table S1. Calibration statistics for NIRS[†] prediction of CP, NDF, ADF, ADL, Ash, and Total Carbon.

Trait	Math treatment[‡]	Calibration R^2	SEC	SECV	SEP	Validation R^2
			---------------g kg^{-1}--------------			
CP	2,8,6,1	0.989	0.228	0.303	0.517	0.982
NDF	4,10,10,1	0.939	1.371	1.563	2.231	0.882
ADF	3,10,10,1	0.955	0.852	1.107	1.164	0.931
ADL	3,5,5,1	0.882	0.373	0.606	0.577	0.805
Ash	3,10,10,1	0.963	0.476	0.662	0.449	0.937
Total Carbon	3,5,5,1	0.918	0.277	0.517	0.192	0.922

[†]Abbreviations: NIRS=near-infrared spectroscopy, CP=crude protein, NDF=neutral detergent fiber, ADF=acid detergent fiber, ADL=acid detergent lignin, SEC=standard error calibration, SECV=standard error cross validation, SEP=Standard Error of Prediction of validation set.

[‡]Math treatments used by NIRS software = derivative number, gap (nm over which derivative is calculated), smooth (number of points over which data is smoothed), second smooth (number of points).
