# Supplementary material for: Characterization of Novel Sorghum brown midrib Mutants from an EMS-Mutagenized Population
Source: G3 (Bethesda). 2014 Sep 2;4(11):2115–24. doi: 10.1534/g3.114.014001 (PMC4232537; doi:10.1534/g3.114.014001)
Supplement: Supporting Information [file supp_g3.114.014001_TableS2.pdf]

Table S2. Midrib phenotype and genotypes of 26 non-*bmr* lines based on results of test crosses with *bmr2*, *bmr6*, and *bmr12* tester lines.

Line	Mutant Phenotype	Tester Line AOK11 *bmr2*	AN603 ATx623 *bmr6*	AN604 ATx623 *bmr12*	Mutant locus
4	WT	WT	WT	WT	
25	WT	WT	WT	WT	
39	WT	-	WT	WT	
40	WT	WT	WT	WT	
41	*bmr*	WT	WT	WEAK	inconclusive
163	WT	WT	WT	WT	
247	WT	WT	WT	WT	
371	WT	WT	WT	WT	
372	WT	WT	WT	WT	
485	WT	WT	WT	WT	
492	WT	WT	WT	WT	
557	WT	WT	WT	WT	
666	WEAK	WT	WT	WT	inconclusive
706	WT	WT	WEAK	WT	inconclusive
924	WT	WT	WT	WT	
934	WT	WT	WT	WT	
1057	WT	WT	WT	WT	
1074	WT	WT	WT	WT	
1402	WT	WT	WT	WT	
1492	WT	WT	WT	WT	
1593	WT	WT	WT	WT	
1605	WT	WT	WT	WT	
1614	WT	WT	WT	WT	
1634	WT	WT	WT	WT	
1668	WT	WT	WT	WT	
1827	WT	WT	WT	WT	

The F_1 individuals, mutant lines, and check lines were visually classified as being *brown midrib* (*bmr*) or wild-type (WT) phenotype (See Materials and Methods). Based upon the test-crosses, loci and alleles were designated, which appear in the far right column.
