# Supplementary material for: Characterization of Novel Sorghum brown midrib Mutants from an EMS-Mutagenized Population
Source: G3 (Bethesda). 2014 Sep 2;4(11):2115–24. doi: 10.1534/g3.114.014001 (PMC4232537; doi:10.1534/g3.114.014001)
Supplement: Supporting Information [file supp_g3.114.014001_TableS3.pdf]

Table S3. Evaluation of the agronomic traits for the mutant lines

	Stand Count (plants/m)			Time to 50% Anthesis (days after planting)			Height (cm)			Yield (T/ha)		
Line	lsmean	Lower	Upper	lsmean	Lower	Upper	lsmean	Lower	Upper	lsmean	Lower	Upper
BTx623^{T}	14.13	10.31	17.94	73.38	52.66	94.09	153.13	135.79	170.46	17.50	16.07	18.94
BTx623^{N}	16.63	12.95	20.30	72.75	52.28	93.22	148.13	128.63	167.62	17.26	14.19	20.34
RTx430	13.13	9.49	16.76	75.75	55.17	96.34	123.75	103.13	144.37	16.63	14.83	18.44
BWheatland	14.75	10.51	18.99	73.88	53.38	94.37	**110.63**	**90.74**	**130.51**	13.71	12.34	15.08
BTx623 *bmr6*	17.38	13.07	21.68	74.00	53.31	94.69	125.00	105.47	144.53	13.66	10.59	16.72
BTx623 *bmr12*	15.75	12.03	19.47	76.63	56.57	96.68	131.88	111.06	152.69	15.72	14.09	17.35
OK11 *bmr2*	15.13	11.48	18.77	78.00	57.39	98.61	120.00	101.98	138.02	**12.02**	**10.09**	**13.94**
bmr2-2	10.00	6.26	13.74	78.88	58.83	98.92	128.13	108.97	147.28	**7.12**	**6.15**	**8.09**
bmr6-23	15.13	11.24	19.01	78.50	58.65	98.35	**96.25**	**79.52**	**112.98**	**5.23**	**4.25**	**6.21**
bmr6-31	10.50	6.85	14.15	77.50	57.40	97.60	**108.13**	**91.65**	**124.60**	**5.20**	**4.23**	**6.17**
bmr6-32	12.88	9.25	16.50	78.00	57.60	98.40	115.63	94.34	136.91	**9.76**	**8.68**	**10.83**
bmr6-45	10.63	7.01	14.24	78.13	57.73	98.52	**114.38**	**94.17**	**134.58**	**6.17**	**5.16**	**7.18**
bmr6-307	**6.25**	**2.47**	**10.03**	81.63	61.29	101.96	**112.50**	**94.06**	**130.94**	**3.48**	**1.99**	**4.96**
bmr6-741	16.50	12.86	20.14	79.50	59.11	99.89	128.13	108.96	147.29	**10.53**	**9.11**	**11.96**
bmr6-971	14.88	11.02	18.73	79.00	58.69	99.31	125.00	105.80	144.20	**11.72**	**10.47**	**12.98**
bmr6-1103	13.13	9.36	16.89	79.13	59.06	99.19	**91.88**	**71.92**	**111.83**	**8.26**	**6.88**	**9.64**
bmr6-1277	10.75	6.40	15.10	76.88	56.53	97.22	**104.38**	**83.20**	**125.55**	**7.95**	**6.66**	**9.24**
bmr12-30	8.50	4.83	12.17	86.63	66.48	106.77	118.75	101.52	135.98	**6.51**	**5.48**	**7.54**
bmr12-34	12.88	8.96	16.79	82.83	62.43	103.23	133.13	115.58	150.67	**8.45**	**7.40**	**9.49**
bmr12-35	16.25	12.48	20.02	74.13	53.57	94.68	132.50	112.12	152.88	**12.95**	**11.51**	**14.38**
bmr12-820	11.21	7.49	14.93	89.37	71.06	107.67	126.83	107.39	146.26	**4.83**	**3.53**	**6.13**
bmr29	12.25	8.33	16.17	75.50	54.86	96.14	133.13	116.21	150.04	**7.30**	**6.34**	**8.27**
bmr30	**5.25**	**0.57**	**9.93**	84.00	64.28	103.72	128.75	111.38	146.12	**4.77**	**3.53**	**6.00**
bmr31	9.21	5.22	13.20	84.87	65.34	104.39	**69.33**	**52.75**	**85.91**	**2.30**	**-0.43**	**5.03**
bmr32-1	7.88	3.84	11.91	82.25	62.52	101.98	126.88	109.42	144.33	**6.91**	**4.97**	**8.86**
bmr32-2	11.88	8.15	15.60	76.13	55.43	96.82	**115.00**	**97.51**	**132.49**	**8.26**	**7.26**	**9.27**
bmr32-3	**6.25**	**2.46**	**10.04**	82.75	63.49	102.01	117.50	97.39	137.61	**3.49**	**1.69**	**5.29**
4	12.25	8.61	15.89	78.13	57.62	98.63	125.63	106.95	144.30	**9.99**	**8.89**	**11.09**
25	11.88	8.23	15.52	87.40	69.54	105.26	148.13	131.07	165.18	**9.75**	**7.53**	**11.96**
39	8.00	4.05	11.95	84.50	63.84	105.16	**100.63**	**82.12**	**119.13**	**6.63**	**5.34**	**7.92**
40	8.63	4.92	12.33	78.75	58.25	99.25	126.88	106.25	147.50	**10.62**	**9.55**	**11.69**
41	13.75	10.03	17.47	75.50	55.20	95.80	116.88	97.06	136.69	**9.71**	**8.71**	**10.70**
163	17.38	13.69	21.06	80.75	60.29	101.21	122.50	102.23	142.77	**8.61**	**7.63**	**9.60**
247	6.71	2.35	11.07	87.20	66.97	107.43	**83.49**	**65.20**	**101.79**	**2.13**	**1.11**	**3.14**
371	14.00	10.19	17.81	79.13	59.04	99.21	149.38	132.40	166.35	15.07	13.29	16.86
372	6.88	2.55	11.20	76.25	56.22	96.28	138.13	118.44	157.81	**12.12**	**10.71**	**13.53**
485	13.38	9.63	17.12	75.75	55.05	96.45	144.38	124.26	164.49	**11.09**	**9.89**	**12.28**
492	10.75	7.11	14.39	85.25	65.89	104.61	**107.50**	**87.33**	**127.67**	**5.89**	**4.93**	**6.84**
557	9.75	6.10	13.40	90.63	70.09	111.16	130.00	113.36	146.64	**7.92**	**6.95**	**8.90**
666	14.75	10.92	18.58	77.75	57.44	98.06	134.38	113.26	155.49	**9.48**	**7.39**	**11.57**

706	7.50	3.85	11.15	84.13	63.88	104.37	**108.75**	**89.78**	**127.72**	**4.28**	**3.10**	**5.46**
924	15.00	11.35	18.65	76.13	55.58	96.67	**99.38**	**80.46**	**118.29**	**9.19**	**7.58**	**10.80**
934	8.25	3.90	12.60	88.38	69.42	107.33	**103.13**	**82.20**	**124.05**	**2.76**	**0.99**	**4.53**
1057	10.38	6.72	14.03	75.38	55.08	95.67	119.38	99.14	139.61	**6.38**	**5.43**	**7.33**
1074	14.25	10.32	18.18	74.88	54.21	95.54	135.00	113.92	156.08	**13.39**	**11.81**	**14.97**
1402	9.75	5.81	13.69	82.38	62.62	102.13	117.50	96.89	138.11	**6.00**	**4.50**	**7.51**
1492	**4.75**	**1.06**	**8.44**	79.50	59.00	100.00	121.25	100.90	141.60	**4.45**	**1.78**	**7.11**
1593	13.50	9.01	17.99	87.71	68.09	107.32	**90.63**	**74.61**	**106.64**	**3.81**	**2.80**	**4.81**
1605	11.13	7.30	14.95	77.63	57.28	97.97	166.25	146.41	186.09	**9.83**	**8.51**	**11.15**
1614	11.00	7.18	14.82	77.13	56.64	97.61	128.75	109.01	148.49	**8.37**	**7.17**	**9.58**
1634	8.13	4.46	11.79	86.63	66.51	106.74	**99.38**	**78.94**	**119.81**	**3.79**	**2.84**	**4.73**
1668	11.13	7.51	14.74	79.38	59.43	99.32	**113.13**	**96.02**	**130.23**	**7.17**	**6.14**	**8.20**
1827	11.50	7.87	15.13	79.00	58.57	99.43	129.38	110.83	147.92	**7.08**	**5.80**	**8.37**

Measurement of these traits is as described in Materials and Methods. Yield is defined as the total above ground biomass. Least Squares Means (lsmean) for mutant and check lines were generated, and means of all variables were ranked from highest (Upper) to lowest (Lower). **Bold text** indicates values that were significantly different ($P \leq 0.20$) from the values of BTx623^{T}, the line used for mutagenesis. Additional details of the statistical analysis are described in Materials and Methods. BTx623^{N} is the line maintained by ARS in Lincoln, NE, which was included for comparison.
