# Supplementary material for: Characterization of Novel Sorghum brown midrib Mutants from an EMS-Mutagenized Population
Source: G3 (Bethesda). 2014 Sep 2;4(11):2115–24. doi: 10.1534/g3.114.014001 (PMC4232537; doi:10.1534/g3.114.014001)
Supplement: Supporting Information [file supp_g3.114.014001_TableS4.pdf]

**Table S4. NIRS prediction for NDF, ADF and ADL of the mutant lines**

| | NDF (%) | | | ADF (%) | | | ADL (%) | | |
|---|---|---|---|---|---|---|---|---|---|
| Line | lsmean | Lower | Upper | lsmean | Lower | Upper | lsmean | Lower | Upper |
| BTx623$^{T}$ | 60.86 | 58.85 | 62.86 | 34.23 | 33.50 | 34.97 | 5.18 | 4.98 | 5.39 |
| BTx623$^{N}$ | 61.48 | 59.48 | 63.49 | 34.52 | 33.76 | 35.28 | 5.34 | 5.13 | 5.54 |
| RTx430 | 64.41 | 62.40 | 66.42 | **36.41** | **35.69** | **37.13** | 5.11 | 4.92 | 5.31 |
| BWheatland | 64.03 | 62.02 | 66.03 | **36.04** | **35.32** | **36.76** | 5.13 | 4.93 | 5.33 |
| BTx623 *bmr6* | 60.76 | 58.75 | 62.76 | 33.76 | 32.97 | 34.54 | **4.28** | **4.07** | **4.49** |
| BTx623 *bmr12* | 62.68 | 60.68 | 64.69 | 34.60 | 33.81 | 35.39 | **4.09** | **3.88** | **4.31** |
| OK11 *bmr2* | **64.79** | **62.78** | **66.79** | 35.53 | 34.79 | 36.27 | **4.05** | **3.85** | **4.25** |
| *bmr2-2* | 58.84 | 56.84 | 60.84 | **31.96** | **31.22** | **32.70** | **3.67** | **3.47** | **3.88** |
| *bmr6-23* | 60.84 | 58.83 | 62.85 | 32.82 | 32.13 | 33.51 | **4.59** | **4.40** | **4.77** |
| *bmr6-31* | 59.10 | 57.09 | 61.11 | **31.19** | **30.49** | **31.89** | **3.77** | **3.58** | **3.97** |
| *bmr6-32* | 61.02 | 59.02 | 63.02 | 33.05 | 32.31 | 33.78 | **3.82** | **3.62** | **4.02** |
| *bmr6-45* | **54.98** | **52.98** | **56.99** | **29.30** | **28.60** | **30.00** | **3.72** | **3.53** | **3.91** |
| *bmr6-307* | 58.15 | 56.14 | 60.15 | **29.13** | **28.35** | **29.91** | **4.30** | **4.09** | **4.51** |
| *bmr6-741* | 59.88 | 57.87 | 61.88 | **32.49** | **31.77** | **33.20** | **3.92** | **3.73** | **4.12** |
| *bmr6-971* | 60.14 | 58.14 | 62.15 | **32.59** | **31.84** | **33.35** | **3.97** | **3.77** | **4.18** |
| *bmr6-1103* | 59.44 | 57.43 | 61.44 | **32.30** | **31.54** | **33.06** | **3.74** | **3.54** | **3.95** |
| *bmr6-1277* | **68.66** | **66.66** | **70.67** | **38.56** | **37.87** | **39.26** | 5.08 | 4.89 | 5.27 |
| *bmr12-30* | 61.02 | 59.02 | 63.03 | **32.40** | **31.64** | **33.16** | **3.85** | **3.64** | **4.05** |
| *bmr12-34* | 61.52 | 59.52 | 63.53 | 33.25 | 32.47 | 34.03 | **3.75** | **3.53** | **3.96** |
| *bmr12-35* | 61.80 | 59.79 | 63.80 | 33.86 | 33.11 | 34.62 | **4.61** | **4.41** | **4.82** |
| *bmr12-820* | 58.46 | 56.44 | 60.49 | **31.12** | **30.25** | **31.98** | **3.37** | **3.13** | **3.60** |
| *bmr29* | 64.41 | 62.41 | 66.42 | **37.32** | **36.58** | **38.06** | **4.65** | **4.45** | **4.85** |
| *bmr30* | 61.29 | 59.28 | 63.30 | **32.31** | **31.51** | **33.12** | **4.50** | **4.28** | **4.72** |
| *bmr31* | 60.07 | 58.06 | 62.08 | **31.48** | **30.67** | **32.29** | 4.80 | 4.58 | 5.02 |
| *bmr32-1* | 62.34 | 60.33 | 64.34 | 33.52 | 32.78 | 34.26 | **4.02** | **3.82** | **4.22** |
| *bmr32-2* | 63.47 | 61.47 | 65.47 | 35.15 | 34.41 | 35.89 | **4.34** | **4.14** | **4.54** |
| *bmr32-3* | 59.89 | 57.89 | 61.90 | **31.37** | **30.63** | **32.10** | **4.53** | **4.33** | **4.74** |
| 4 | 64.05 | 62.05 | 66.06 | **35.76** | **35.01** | **36.52** | 5.51 | 5.30 | 5.71 |
| 25 | 56.90 | 54.90 | 58.91 | **30.60** | **29.79** | **31.41** | **3.64** | **3.42** | **3.86** |
| 39 | 61.21 | 59.20 | 63.22 | 32.82 | 32.01 | 33.62 | 4.99 | 4.77 | 5.21 |
| 40 | 62.45 | 60.44 | 64.45 | 34.33 | 33.57 | 35.09 | 5.47 | 5.26 | 5.68 |
| 41 | 60.85 | 58.84 | 62.85 | 33.14 | 32.38 | 33.90 | 5.09 | 4.89 | 5.30 |
| 163 | 62.53 | 60.53 | 64.54 | 34.63 | 33.87 | 35.38 | **4.77** | **4.56** | **4.98** |
| 247 | 61.51 | 59.50 | 63.53 | 32.73 | 31.90 | 33.57 | 5.20 | 4.97 | 5.43 |
| 371 | **64.94** | **62.93** | **66.94** | **36.61** | **35.86** | **37.37** | 5.41 | 5.21 | 5.62 |
| 372 | 60.40 | 58.39 | 62.40 | **32.72** | **31.96** | **33.48** | 5.00 | 4.79 | 5.20 |
| 485 | 59.26 | 57.26 | 61.27 | 32.88 | 32.15 | 33.62 | **4.47** | **4.27** | **4.68** |
| 492 | 58.94 | 56.93 | 60.95 | **31.68** | **30.87** | **32.48** | **4.29** | **4.07** | **4.51** |
| 557 | 59.61 | 57.61 | 61.62 | **31.87** | **31.11** | **32.63** | 4.78 | 4.57 | 4.98 |
| 666 | **65.34** | **63.33** | **67.34** | **36.09** | **35.37** | **36.81** | **4.10** | **3.90** | **4.30** |

| | | | | | | | | | |
|---|---|---|---|---|---|---|---|---|---|
| 706 | 59.88 | 57.87 | 61.88 | **30.63** | **29.89** | **31.37** | 5.58 | 5.38 | 5.78 |
| 924 | **66.00** | **64.00** | **68.01** | **37.53** | **36.79** | **38.27** | **5.84** | **5.64** | **6.04** |
| 934 | 62.82 | 60.81 | 64.82 | 33.45 | 32.73 | 34.16 | 5.07 | 4.87 | 5.26 |
| 1057 | 61.66 | 59.66 | 63.67 | 33.60 | 32.86 | 34.33 | **4.71** | **4.51** | **4.91** |
| 1074 | 59.80 | 57.80 | 61.81 | 32.92 | 32.16 | 33.68 | **4.75** | **4.54** | **4.96** |
| 1402 | 57.51 | 55.51 | 59.52 | **30.97** | **30.21** | **31.73** | **4.23** | **4.02** | **4.44** |
| 1492 | 60.98 | 58.97 | 62.99 | **32.48** | **31.67** | **33.28** | 4.93 | 4.71 | 5.16 |
| 1593 | **55.73** | **53.72** | **57.74** | **29.59** | **28.91** | **30.27** | **4.14** | **3.95** | **4.32** |
| 1605 | 57.65 | 55.64 | 59.66 | **32.27** | **31.59** | **32.95** | 5.02 | 4.84 | 5.21 |
| 1614 | 62.41 | 60.41 | 64.41 | 34.75 | 34.00 | 35.51 | **5.72** | **5.51** | **5.92** |
| 1634 | 57.59 | 55.59 | 59.59 | **28.78** | **28.03** | **29.54** | 4.80 | 4.59 | 5.01 |
| 1668 | 59.55 | 57.55 | 61.56 | **31.57** | **30.79** | **32.35** | **4.70** | **4.49** | **4.91** |
| 1827 | **56.55** | **54.55** | **58.55** | **30.66** | **29.92** | **31.39** | **4.05** | **3.85** | **4.25** |

Values for neutral detergent fiber (NDF), acid detergent fiber (ADF), and acid detergent lignin (ADL) concentration were determined by NIRS prediction based on the calibration equation (Table S1). Additional details are found in the Materials and Methods Section. Least Squares Means (lsmean) for mutant and check lines were generated, and means of all variables were ranked from highest (Upper) to lowest (Lower). **Bold text** indicates values that were statistically significantly different ($P \leq 0.20$) from the values of BTx623$^{T}$, the line used for mutagenesis. Additional details of the statistical analysis are described in the Materials and Methods section. BTx623$^{N}$ is the line maintained by ARS in Lincoln, NE, which was included for comparison.
