# Supplementary material for: Characterization of Novel Sorghum brown midrib Mutants from an EMS-Mutagenized Population
Source: G3 (Bethesda). 2014 Sep 2;4(11):2115–24. doi: 10.1534/g3.114.014001 (PMC4232537; doi:10.1534/g3.114.014001)
Supplement: Supporting Information [file supp_g3.114.014001_TableS5.pdf]

Table S5. NIRS prediction for Total Ash, Crude Protein and Total Carbon of the *bmr* mutant lines.

	Ash (%)			Crude Protein (%)			Total Carbon (%)		
Line	lsmean	Lower	Upper	lsmean	Lower	Upper	lsmean	Lower	Upper
BTx623[T]	11.60	11.13	12.06	5.46	4.19	6.73	43.28	43.09	43.47
BTx623[N]	11.72	11.25	12.20	5.76	4.50	7.02	43.29	43.09	43.48
RTx430	10.94	10.49	11.39	6.86	5.58	8.14	**44.03**	**43.84**	**44.21**
BWheatland	12.43	11.97	12.88	7.21	5.94	8.49	43.06	42.87	43.24
BTx623 *bmr6*	**14.52**	**14.03**	**15.01**	6.10	4.85	7.36	**41.85**	**41.65**	**42.05**
BTx623 *bmr12*	12.12	11.63	12.62	6.95	5.70	8.21	43.22	43.02	43.42
OK11 *bmr2*	**12.59**	**12.13**	**13.06**	6.66	5.39	7.93	**42.82**	**42.63**	**43.01**
bmr2-2	11.14	10.68	11.61	5.30	4.03	6.56	43.31	43.12	43.50
bmr6-23	12.28	11.84	12.71	7.19	5.90	8.49	43.03	42.85	43.21
bmr6-31	11.33	10.89	11.77	7.59	6.31	8.88	43.19	43.01	43.37
bmr6-32	10.85	10.38	11.31	6.98	5.71	8.25	43.63	43.45	43.82
bmr6-45	**10.59**	**10.15**	**11.03**	5.90	4.62	7.19	43.24	43.06	43.42
bmr6-307	**13.26**	**12.77**	**13.75**	**8.48**	**7.22**	**9.74**	**42.48**	**42.28**	**42.68**
bmr6-741	11.54	11.09	11.99	6.03	4.75	7.30	43.12	42.93	43.31
bmr6-971	11.91	11.43	12.39	6.32	5.06	7.58	43.00	42.80	43.19
bmr6-1103	11.66	11.19	12.14	6.21	4.95	7.47	43.04	42.85	43.24
bmr6-1277	**13.33**	**12.89**	**13.77**	7.03	5.74	8.31	43.17	42.98	43.35
bmr12-30	10.78	10.30	11.26	**8.04**	**6.78**	**9.30**	**43.71**	**43.51**	**43.90**
bmr12-34	**9.76**	**9.27**	**10.25**	6.58	5.32	7.83	**44.10**	**43.90**	**44.30**
bmr12-35	**10.26**	**9.78**	**10.74**	5.22	3.96	6.48	**43.94**	**43.75**	**44.13**
bmr12-820	**9.85**	**9.31**	**10.39**	7.57	6.32	8.81	43.68	43.46	43.89
bmr29	10.81	10.34	11.27	6.21	4.95	7.48	**43.83**	**43.64**	**44.02**
bmr30	**10.53**	**10.02**	**11.03**	7.63	6.38	8.88	**43.84**	**43.64**	**44.04**
bmr31	11.22	10.72	11.72	**9.72**	**8.47**	**10.98**	43.27	43.07	43.47
bmr32-1	**7.92**	**7.45**	**8.38**	5.22	3.95	6.50	**44.65**	**44.46**	**44.84**
bmr32-2	**9.86**	**9.39**	**10.32**	5.89	4.62	7.16	**44.09**	**43.90**	**44.28**
bmr32-3	**10.43**	**9.96**	**10.89**	**8.23**	**6.96**	**9.49**	**43.77**	**43.58**	**43.96**
4	11.50	11.02	11.98	7.20	5.94	8.46	43.47	43.28	43.67
25	**7.41**	**6.90**	**7.91**	4.97	3.72	6.22	**44.48**	**44.28**	**44.68**
39	**10.49**	**9.98**	**10.99**	7.83	6.58	9.08	**43.94**	**43.73**	**44.14**
40	12.23	11.75	12.70	6.93	5.67	8.19	43.28	43.08	43.47
41	10.80	10.32	11.27	6.11	4.86	7.37	43.47	43.27	43.66
163	**10.18**	**9.70**	**10.66**	6.03	4.77	7.29	**43.99**	**43.80**	**44.18**
247	12.14	11.62	12.66	**9.94**	**8.69**	**11.19**	43.39	43.18	43.60
371	11.12	10.64	11.59	5.00	3.74	6.26	43.59	43.40	43.78
372	11.36	10.89	11.84	6.35	5.09	7.61	43.40	43.21	43.59
485	**10.14**	**9.67**	**10.60**	4.47	3.20	5.74	43.49	43.31	43.68
492	**9.31**	**8.81**	**9.82**	6.60	5.35	7.85	**44.14**	**43.94**	**44.34**

557	10.87	10.40	11.35	7.48	6.22	8.74	43.48	43.28	43.67
666	**8.29**	**7.84**	**8.75**	4.95	3.67	6.23	**44.87**	**44.68**	**45.05**
706	11.07	10.61	11.54	7.68	6.42	8.95	43.20	43.01	43.39
924	12.18	11.72	12.65	7.27	6.00	8.54	**43.70**	**43.51**	**43.89**
934	11.70	11.25	12.15	**9.04**	**7.77**	**10.32**	43.46	43.27	43.64
1057	11.63	11.16	12.09	7.03	5.77	8.30	43.13	42.94	43.32
1074	11.24	10.77	11.72	5.90	4.64	7.16	43.20	43.01	43.39
1402	**9.65**	**9.17**	**10.13**	6.31	5.05	7.57	43.66	43.47	43.86
1492	10.67	10.16	11.17	6.96	5.71	8.21	43.63	43.43	43.84
1593	**10.31**	**9.88**	**10.74**	**9.06**	**7.76**	**10.35**	43.60	43.42	43.78
1605	**9.82**	**9.39**	**10.25**	6.13	4.83	7.42	**43.85**	**43.67**	**44.03**
1614	12.12	11.65	12.60	6.80	5.54	8.06	43.14	42.94	43.33
1634	**10.53**	**10.05**	**11.00**	7.41	6.15	8.67	43.53	43.34	43.73
1668	**10.24**	**9.75**	**10.73**	7.24	5.98	8.49	43.66	43.46	43.85
1827	**9.63**	**9.16**	**10.09**	5.14	3.87	6.41	**43.73**	**43.54**	**43.92**

Values for ash, crude protein and total carbon were determined by NIRS prediction based on the calibration equation (Table S1). Additional details are found in the Materials and Methods Section. Least Squares Means (lsmean) for mutant and check lines were generated, and means of all variables were ranked from highest (Upper) to lowest (Lower). **Bold text** indicates values that were statistically significantly different ($P \leq 0.20$) from the values of BTx623^{T}, the line used for mutagenesis. Additional details of the statistical analysis are described in the Materials and Methods section. BTx623^{N} is the line maintained by ARS in Lincoln, NE, which was included for comparison.
