# Supplementary material for: Characterization of Novel Sorghum brown midrib Mutants from an EMS-Mutagenized Population
Source: G3 (Bethesda). 2014 Sep 2;4(11):2115–24. doi: 10.1534/g3.114.014001 (PMC4232537; doi:10.1534/g3.114.014001)
Supplement: Supporting Information [file supp_g3.114.014001_FigureS1.pdf]

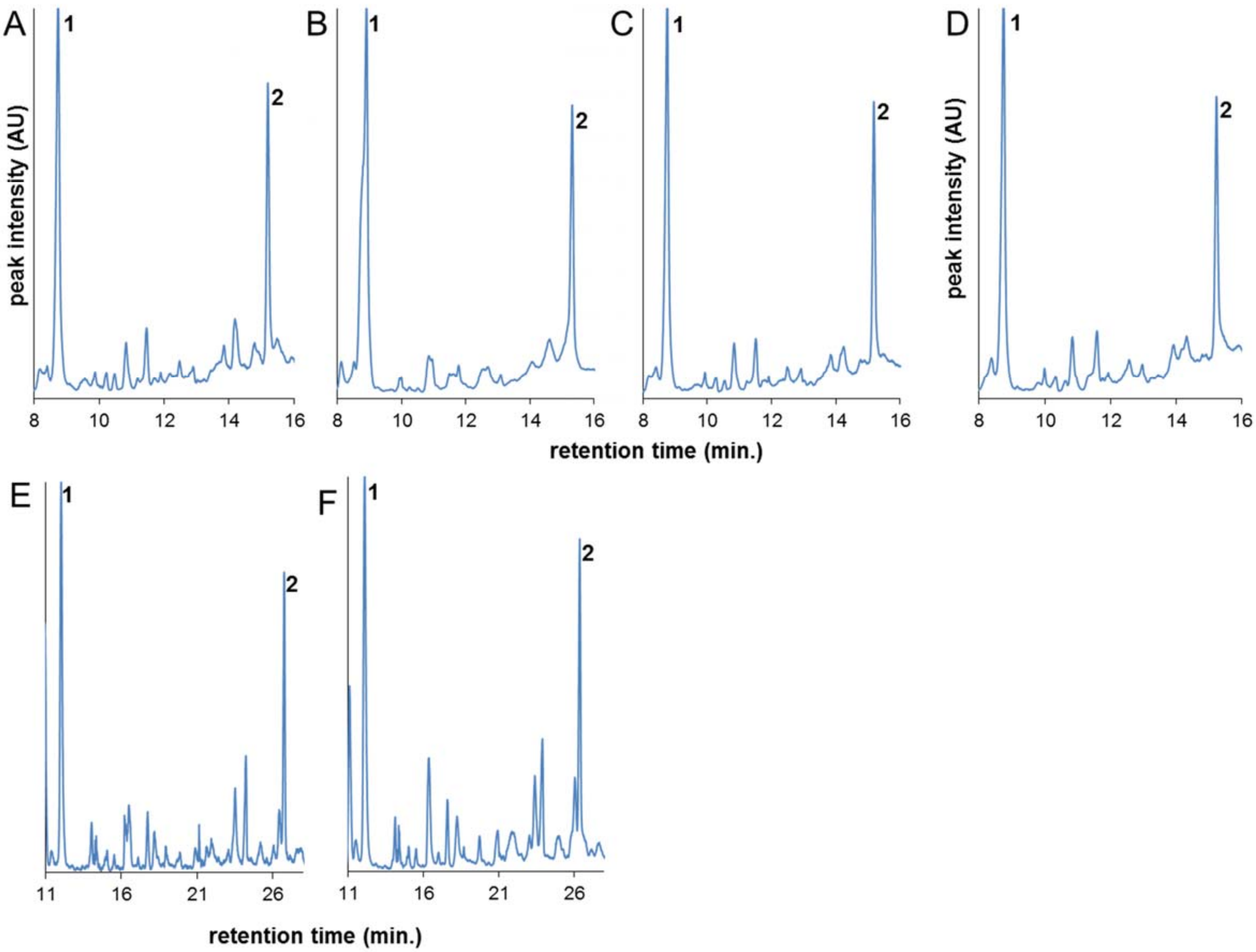

Figure S1 Partial pyrograms obtained with stover from **A.** BTx623, **B.** *bmr30*, **C.** *bmr31*, **D.** *bmr32-1*, **E.** BTx623, and **F.** *bmr29*. Peaks **1** and **2** represent 2-methoxy-4-methyl phenol (*m*/*z* 138, 123; derived from guaiacyl residues) and 2,6-dimethoxy-4-methyl phenol (*m*/*z* 168, 153; derived from syringyl residues). The difference in retention times between A-D and E, F reflects the slower ramp rate during gas chromatography of the latter two samples, to improve resolution.
